# Site-selected thionated benzothioxanthene chromophores as heavy-atom-free small-molecule photosensitizers for photodynamic therapy

**DOI:** 10.1038/s42004-022-00752-x

**Published:** 2022-10-31

**Authors:** Marco Deiana, Pierre Josse, Clément Dalinot, Artem Osmolovskyi, Pablo Simón Marqués, José María Andrés Castán, Laura Abad Galán, Magali Allain, Lhoussain Khrouz, Olivier Maury, Tangui Le Bahers, Philippe Blanchard, Sylvie Dabos-Seignon, Cyrille Monnereau, Nasim Sabouri, Clément Cabanetos

**Affiliations:** 1 Department of Medical Biochemistry and Biophysics, Umeå University, SE-901 87 Umeå, Sweden; 2 Univ Angers, CNRS, MOLTECH-ANJOU, SFR MATRIX, F-49000 Angers, France; 3 Univ Lyon, ENS de Lyon, CNRS UMR 5182, Université Claude Bernard Lyon 1, F-69342 Lyon, France; 4 IRL CNRS 2002, 2BFUEL, CNRS -Yonsei University, Seoul, South Korea

**Keywords:** Photobiology, Drug discovery and development

## Abstract

Photodynamic therapy is a clinically approved anticancer modality that employs a light-activated agent (photosensitizer) to generate cytotoxic reactive oxygen species (ROS). There is therefore a growing interest for developing innovative photosensitizing agents with enhanced phototherapeutic performances. Herein, we report on a rational design synthetic procedure that converts the ultrabright benzothioxanthene imide (**BTI**) dye into three heavy-atom-free thionated compounds featuring close-to-unit singlet oxygen quantum yields. In contrast to the **BTI**, these thionated analogs display an almost fully quenched fluorescence emission, in agreement with the formation of highly populated triplet states. Indeed, the sequential thionation on the **BTI** scaffold induces torsion of its skeleton reducing the singlet-triplet energy gaps and enhancing the spin-orbit coupling. These potential PSs show potent cancer-cell ablation under light irradiation while remaining non-toxic under dark condition owing to a photo-cytotoxic mechanism that we believe simultaneously involves singlet oxygen and superoxide species, which could be both characterized in vitro. Our study demonstrates that this simple site-selected thionated platform is an effective strategy to convert conventional carbonyl-containing fluorophores into phototherapeutic agents for anticancer PDT.

## Abstract (web summary)

Site-selective thionation is a powerful strategy to access heavyatom-free small-molecule photosensitizers for photodynamic therapy. Here, a series of sulfur-substituted benzothioxanthene chromophores is synthesized and the sequential insertion of sulfur atoms is shown to result in strong and concomitant optimization of both light absorption and therapeutic activity.

## Introduction

Cancer pharmacotherapy is often severely hindered by issues related to poor targeting of the molecular signatures of the cancer type and off-target toxicities^1,2^. Conversely, photo-therapeutic modalities rely on the delivery and/or activation of drugs at diseased sites while sparing non-malignant cells^3–6^.

Light offers unparalleled advantages as a non-invasive regulatory element, including low or negligible toxicity, superior spatial and temporal resolution, and the ability to elicit drug activation by adjusting the wavelength, power, and duration of the irradiation^2,7–11^.

The utility of phototherapies is highlighted by the success of photodynamic therapy (PDT)^3,12–14^. PDT involves the combination of a photosensitizing agent (PS) and light that together generate cytotoxic reactive singlet oxygen (^1^O_2_) that precisely destroys the cancer cells at the targeted tumor sites^15–19^.

The continuous development in light delivery technologies^20^ coupled with the rational design of new and efficient PSs^15^ that absorb in the far-red and NIR^21–24^ provide the means to influence cancer biology with an even more exquisite precision.

However, such PSs sometimes feature heavy atoms to foster their triplet yields, causing concerns about dark cytotoxicity, potential high costs, and the difficult of synthesis of such metal complexes^25–27^.

Alternatively, large polyaromatic structures (porphyrins or related derivatives) are most often used in clinical treatments, at the cost of often costly and tedious synthetic procedures, and sometimes significant long-term toxicity because of slow metabolization processes^15^.

In view of these drawbacks, there is an urgent need to develop new and synthetically accessible heavy-atom-free PSs to better fit the clinical requirements for PDT-mediated cancer treatments (in terms of site-selective accumulation, absorption properties in the biological transparency window, and singlet oxygen photosensitization efficiency).

Thus far, various molecular engineering strategies devoted to design metal-free PSs have been reported^15,28^. The recent burst of reports with structural variations of the “BODIPY” core combining intense absorption in the red and high singlet oxygen generation efficiency for PDT applications is a particularly significant illustration of the interest of such approaches^29–36^.

Site-selected thionation in the PS´ scaffold is another even more recent approach used to impart unique structural, photochemical, and therapeutic outcomes^26,37–41^. Indeed, Yoon´s group reported on the use of thionaphthalimides as efficient ROS generators under both normoxia and hypoxia conditions^37^. Even more recently, Chou and co-workers reported on the use of thione-derived perylene diimides as promising PDT agents^41^.

Central to this progress, we report herein a simple and efficient method that converts the ultrabright benzothioxanthene imide (**BTI**) fluorophore into mono- (hereafter, **T1** and **T2**) and di- (henceforth, **T3**) thionated heavy-atom-free PSs. Site-selected thionation on the **BTI** scaffold induces a remarkable fluorescence quenching that is accompanied by an impressive increase of the singlet oxygen quantum yield that reaches a close-to-unit value. Theoretical studies reveal that the sulfur substitution of the carbonyl groups in the **BTI** structure enhances the spin orbit coupling (SOC) owing to a dual effect: it lowers the level of the singlet n→π* excited state which decreases the singlet-triplet energy gaps, and induces a torsion of its skeleton, relaxing selection rules for the intersystem crossing. These last features are indeed responsible for the efficient population of the triplet state and for the unique spectroscopic and photophysical properties of **T1**–**T3**. More importantly, we demonstrated that all compounds exert minimal dark cytotoxicity while becoming highly phototoxic at nanomolar concentrations under light irradiation thus exhibiting very large phototherapeutic indices.

## Results and discussion

### Synthesis and structural characterization

The synthesis of **T1**, **T2**, and **T3** was extremely straightforward. It could be achieved simply by treating the *N*-alkylated **BTI**, which seminal three steps synthesis^42,43^ was recently updated^44^, with 2 molar equivalents of Lawesson’s reagent as depicted in Fig. 1 (for NMR and HRMS spectra see Supplementary Figs. 1–10).

**Fig. 1:**
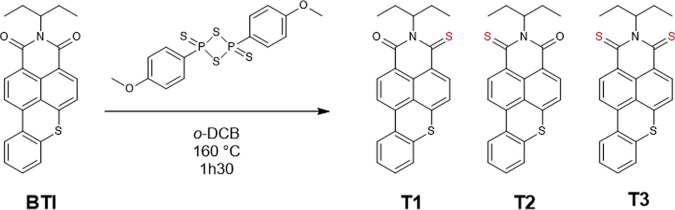
Synthesized compounds. Thionation reaction of **BTI** afforded compounds **T1**–**T3**.

**Fig. 2:**
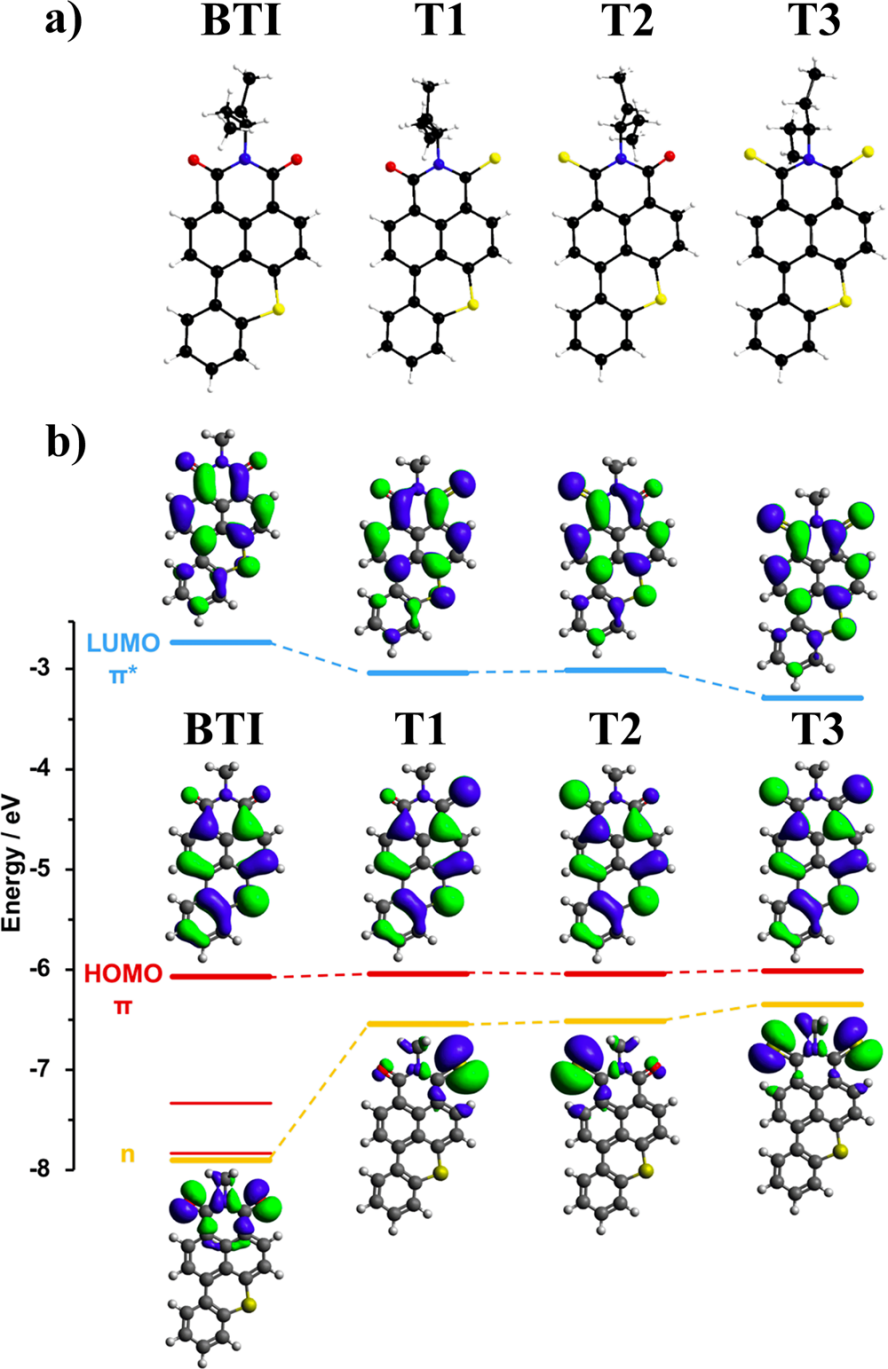
X-ray structures and computed data for T1-T3. **a** Molecular structures of **BTI** and the related thionated compounds (**T1**–**T3**) confirmed by X-ray diffraction on single crystals (S: yellow, O: red, C: black, H: gray, N: blue). **b** DFT computed molecular orbitals levels (PBE0/6-311 + G(d,p)/dichloromethane PCM). Occupied π orbitals, occupied *n* orbitals and unoccupied π* orbitals are in red, orange, and blue, respectively. Orbital iso-surfaces are drawn with an iso-contour of 0.02 a.u.

In order to determine the degree of sulfur substitution and, in the case of mono-substituted products, the identity of both regioisomers, the three isolated compounds **T1**, **T2** and **T3** were subjected to a set of structural characterizations. High resolution mass spectrometry (HRMS) measurements were thus first carried out revealing that **T3** actually corresponds to the di-thionated derivative and both **T1** and **T2** to mono-thionated species (Supplementary Figs. 7–9). Analyzed by nuclear magnetic resonance spectroscopy (NMR), structures were first deduced from the peculiar signatures and chemical shifts of the naphthalene ring constituting protons (Supplementary Fig. 10) and finally confirmed by X-ray diffraction since crystals of all isolated thionated compounds were successfully grown by the slow evaporation method (Fig. 2a, Supplementary Table 1 and Supplementary Fig. 11).

X-ray structures highlighted that the replacement of a carbonyl by a thiocarbonyl leads, in all cases, to a significant elongation of the C=S bond length compared to the initial C=O, from *ca* 1.281 Å to up to *ca* 1.672 Å, in consistency with early reported studies^45^. Comparison of crystal structures first revealed that both **BTI** and **T1** molecules crystallize in triclinic P-1 space group with similar cell parameters. The π-conjugated cores are planar and stack along the *a* axis in a head-to-tail manner with distances ranging from 3.477 to 3.569 Å for **BTI** and 3.464 to 3.595 Å for **T1**. The di-thionated derivative, namely **T3**, was also found to crystallize in P-1 space group but with a double c cell parameter (*vs* **BTI**) showing two independent molecules in the asymmetric unit. The latter stack along the *a* axis in a head-to-tail manner with distances of ca 3.387 to 3.469 Å for one molecule and 3.511 to 3.534 Å for the second one. In addition, even if the benzothioxanthene core still remains planar, the thiocarbonyl constituting sulfur atoms deviate from the *π*-conjugated plane of *ca* 0.14–0.76 Å. Finally, and compared to the series, **T2** was found to crystallize in monoclinic P2_1_/c space group. In this case, backbones alternate along the *c* axis with a deviation of *ca* 36° and distances of 3.507 Å.

### State assignment, electrochemical characterization, optical measurements, and spin–orbit coupling studies

Computed data suggested an incremental reduction of the gap depending on the degree of thiocarbonyl substitution, resulting from the stabilization of the lowest unoccupied energy level (LUMO) and destabilization of the highest occupied molecular orbital (HOMO). Interestingly, only the LUMO levels were found to be affected by the second conversion of the carbonyl groups into a thioamide (from **T1**/**T2** to **T3**). Furthermore, computed data predicted only minor effects on the energy levels of the frontiers orbitals of the mono-thionated derivatives (**T1** *vs* **T2**) when varying the position of the thiocarbonyl group (Fig. 2b).

**Fig. 3:**
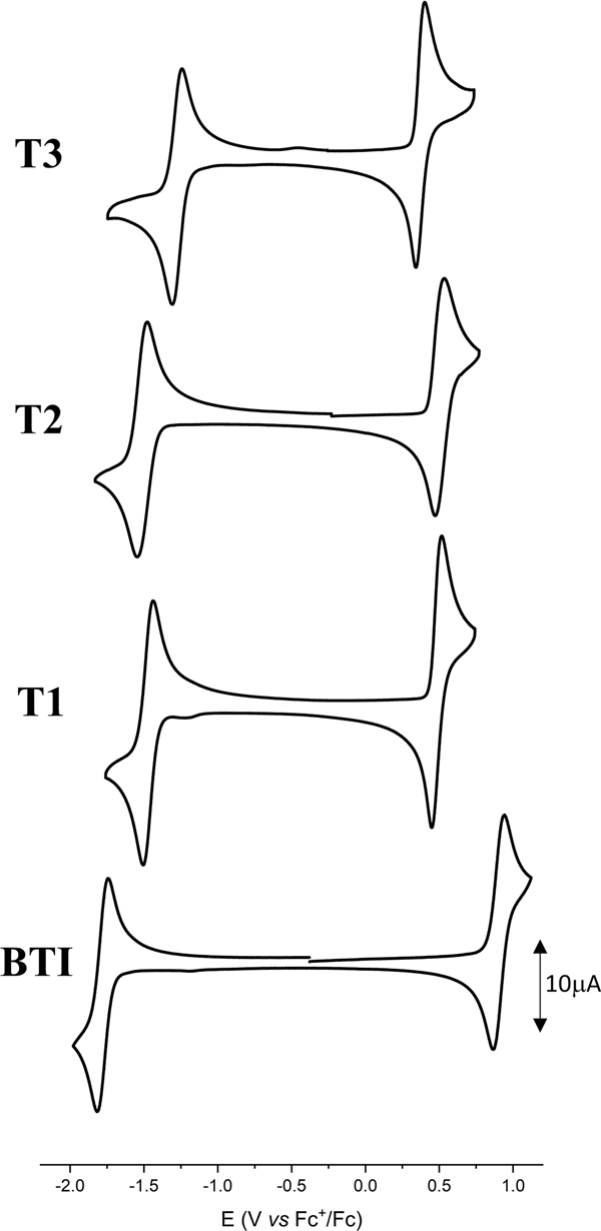
Cyclic voltammetry. Cyclic voltammograms of thionated compounds (**T1**–**T3**) recorded at 100 mV s^−1^ in 0.10 M Bu_4_NPF_6_/CH_2_Cl_2_ and comparison with that of the reference **BTI** compound.

These simulated data were then experimentally tested through the electrochemical characterization of the series. The recorded cyclic voltammograms, plotted in Fig. 3 and the extracted electrochemical data, gathered in Table 1, were found to be fully in line with computed values. As predicted, the successive thionations of **BTI** results in the reduction of the electrochemical band gap without affecting the specific dual redox properties of this class of dyes, since the reversibility of both oxidation and reduction patterns are maintained in the whole series. However, while similar HOMO levels were simulated for all thionated compounds, electrochemistry also confirmed that the number of the thiocarbonyl groups was the dominant factor affecting the energy of the frontier orbitals, their position having comparatively milder effects.

**Table 1:** Electrochemical data performed in dichloromethane, using the tetrabutylammonium hexafluorophosphate (Bu_4_NPF_6_) as supporting electrolyte.

Compound	*E*_pa_ (V)	*E*_pc_ (V)	*E*^ox^_onset_ (V)	*E*^red^_onset_ (V)	HOMO (eV)	LUMO (eV)	Electrochemical band gap (eV)
**T3**	0.45	−1.26	0.37	−1.14	−5.47	−3.96	1.51
**T2**	0.52	−1.50	0.41	−1.38	−5.51	−3.72	1.79
**T1**	0.54	−1.55	0.42	−1.39	−5.52	−3.71	1.81
**BTI**	0.94	−1.81	0.84	−1.69	−5.94	−3.41	2.53

*E*_HOMO_ (eV) = −(*E*^ox^_onset_ + 5.1), *E*_LUMO_ (eV) = −(*E*^red^_onset_+ 5.1).

**Fig. 4:**
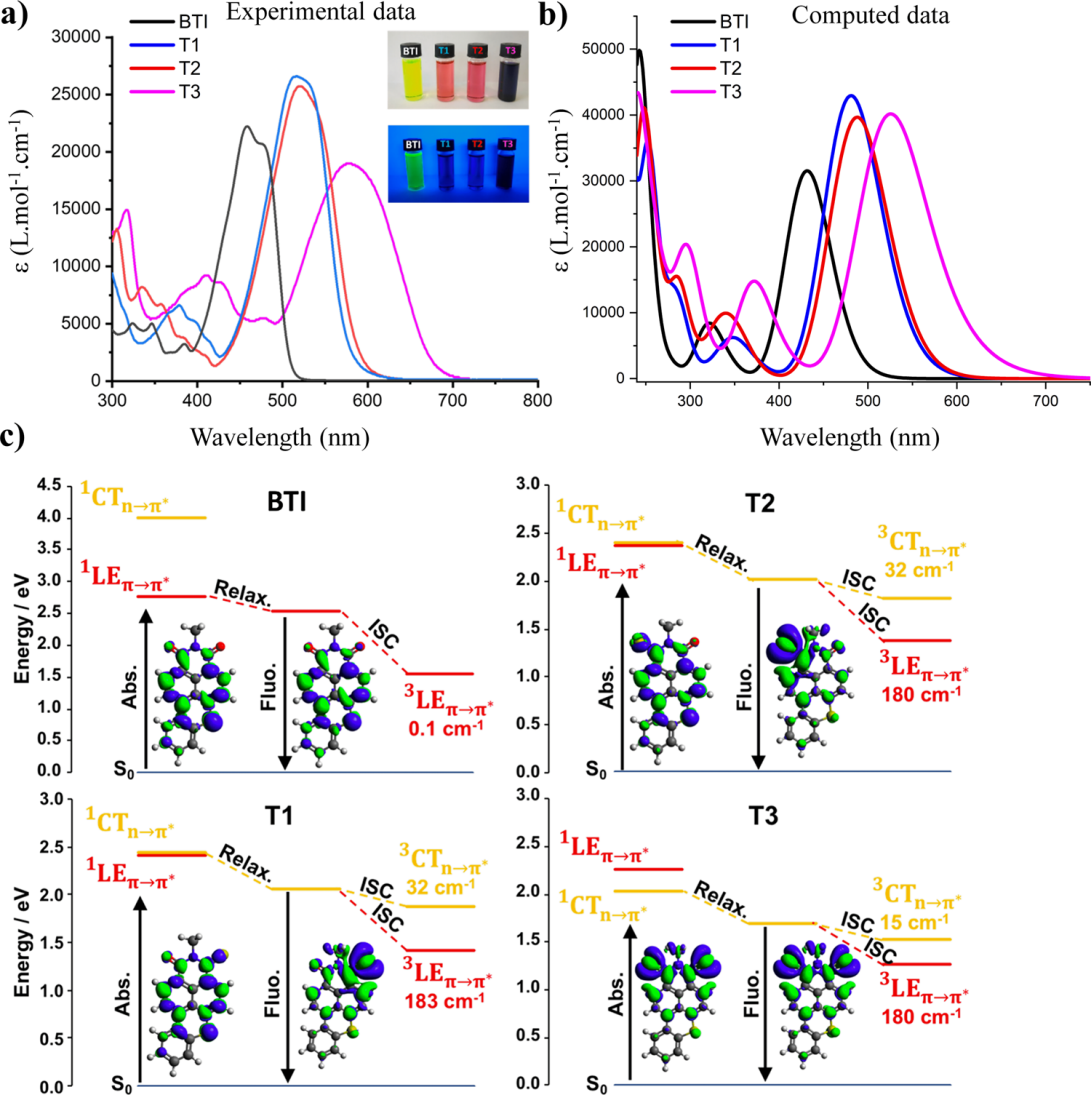
Experimental and computed photophysical properties. **a** UV-visible spectra recorded in solution (Dichloromethane) for **BTI** (black line), **T1** (blue line), **T2** (red line), and **T3** (pink line). Inset: photographs of the solutions under natural (top) and blue light (bottom) to show the absence of emissive properties of **T1**−**T3**. **b** Simulated UV-visible spectra (TD-DFT/OT-ωB97-XD/6-31 G(d,p) /Dichloromethane, *ε* = 8.9) for **BTI** (black line), **T1** (blue line), **T2** (red line) and **T3** (pink line). **c** TD-DFT computed electronic structure of **BTI** and **T1**−**T3**. The variation of electron density is given for the S_0_−S_1_ transition both in absorption and emission considering always S_0_ as the reference state. Blue and green areas correspond to electron depletion and increase, respectively (isosurface 0.001 a.u.). The magnitude of the SOC between S_1_ and the triplet states is given below the triplet state positions in cm^−1^.

These features were also thereafter highlighted by optical measurements (Fig. 4a). Beyond the expected bathochromic shift induced by the successive thionations of the imide on the lowest energy absorption bands, the onset of **T2** absorption indeed appeared slightly redshifted compared to that of **T1** (*E*_*g*_^Opt^ ≈ 0.04 eV), in consistency with the *ca* 0.03 eV difference of electrochemical band gap calculated between these two mono-thionated derivatives. Compared to **BTI**, the replacement of one of the imide constituting oxygen atoms by a sulfur atom (**T1** and **T2**) led to the enhancement of molar absorption coefficients whereas the opposite effect were monitored for **T3**. In this case, the 600 nm centered absorption band is significantly broadened with a decrease of the molar extinction coefficient at the absorption maximum (Table 2)^39^. Interestingly, this spectroscopic study points out that dramatic red-shifts of the absorption wavelengths can be achieved upon thionation of the parent **BTI** molecules, resulting in molecules that efficiently absorb in the far-red region of the electromagnetic spectrum, ideally suited for PDT applications. Regarding the emission properties, it appeared that the fluorescence of the thionated derivatives was totally quenched (inset Fig. 4a). This feature was indeed anticipated since the thionation of dyes is an efficient reported strategy to foster singlet-to-triplet Inter-System Crossings (ISCs).

**Table 2:** Optical Data recorded at room temperature in dichloromethane for **BTI**, **T1**, **T2**, and **T3** unless otherwise mentioned.

Compound	λ_max_ (nm)	*ε* (M^−1^ cm^−1^)	λ_onset_ (nm)	*E*_*g*_^opt^ (eV)	λ_em_ (nm)	φ_F_^a^	τ_obs_ (ns)	φ_Δ_^b^
**BTI**	455	22200	510	2.43	502	0.99	7.48	0
**T1**	508	26600	580	2.13	−	−	−	1.00
**T2**	513	25700	592	2.09	−	−	−	0.98
**T3**	571	19000	684	1.81	−	−	−	0.78

^a^Measured using Coumarine-153 as reference (φ_F_ = 0.45 in MeOH).

^b^Optical data recorded in deuterated chloroform, Phenalenone used as a reference (φΔ = 0.95 in chloroform).

Hence to support this hypothesis and provide a quantum chemical perspective on the electronic structure of these new molecules at the origin of this phenomenon, TD-DFT calculations were performed. As depicted in Fig. 4b, c, it is noteworthy that TD-DFT well reproduces the experimental trend consisting of the gradual red-shift of the absorption band upon sulfur substitution or when moving from **T1** to **T2** (515 and 522 nm, respectively). When compared to **BTI**, the increase in energy of the n-type orbital is reflected in the electronic states of the thionated compound by a n→π* transition extremely close to the HOMO → LUMO π → π* transition. It is noteworthy that the n→π* transition was even found to be the most stable one in the case of **T3**. This phenomenon is also observed in S-based Nile Red derivatives where the incorporation of S atoms makes the π → π* and n→π* extremely close in energy^46^. The charge transfer indexes DCT computed both for the HOMO → LUMO π → π* and the n→π* transitions are around 1.3 and 2.5 Å, respectively, justifying the notation ${{{{{{\rm{LE}}}}}}}_{{{{{{\rm{\pi }}}}}}\to {{{{{{\rm{\pi }}}}}}}^{* }}$ and ${{{{{{\rm{CT}}}}}}}_{{{{{{\rm{n}}}}}}\to {{{{{{\rm{\pi }}}}}}}^{* }}$to label these transitions. For all thionated compounds, the excited state relaxation always converged toward the ${{{{{{\rm{CT}}}}}}}_{{{{{{\rm{n}}}}}}\to {{{{{{\rm{\pi }}}}}}}^{* }}$ as the most stable excited state with an oscillator strength for the return to the S_0_ almost equal to 0, which is in consistent with the weak fluorescence efficiency. SOC between the singlet ${}^{1}{{{{{\rm{CT}}}}}}_{{{{{{\rm{n}}}}}}\to {{{{{{\rm{\pi }}}}}}}^{* }}$ and the two triplet states at lower energies, namely the ${}^{3}{{{{{\rm{LE}}}}}}_{{{{{{\rm{\pi }}}}}}\to {{{{{{\rm{\pi }}}}}}}^{* }}$ and ${}^{3}{{{{{\rm{CT}}}}}}_{{{{{{\rm{n}}}}}}\to {{{{{{\rm{\pi }}}}}}}^{* }}$ states, were thereafter computed. Coupling between the ${}^{1}{{{{{\rm{CT}}}}}}_{{{{{{\rm{n}}}}}}\to {{{{{{\rm{\pi }}}}}}}^{* }}$ and ${}^{3}{{{{{\rm{LE}}}}}}_{{{{{{\rm{\pi }}}}}}\to {{{{{{\rm{\pi }}}}}}}^{* }}$ was found to be extremely large (above 100 cm^−1^) in agreement with the El-Sayed rule stating that Inter-System Crossing processes between n→π* and π→π* transitions are symmetry allowed and also computed for other S-containing organic molecules^46^. Interestingly, even the coupling between ${}^{1}{{{{{\rm{CT}}}}}}_{{{{{{\rm{n}}}}}}\to {{{{{{\rm{\pi }}}}}}}^{* }}$ and ${}^{3}{{{{{\rm{CT}}}}}}_{{{{{{\rm{n}}}}}}\to {{{{{{\rm{\pi }}}}}}}^{* }}$ appeared unexpectedly large (above 10 cm^−1^) which appears this time contradictory to El-Sayed’s rules. In line with our previous works dealing with the origin of SOC in **BTI** derivatives, this behavior can be correlated to the torsion of the **BTI** skeleton at the S_1_ geometry (around 10–15° of torsional angle) for all thionated compounds. This alleviates the symmetry-based restriction rules for the SOC^47,48^. With this theoretical confirmation that the “dark” nature of their singlet electronic excited state can be linked for the three compounds to a very efficient ISC process to a triplet excited state, experimental measurements of singlet oxygen quantum yields were consequently carried out.

### Photosensitization pathways and photostability studies

Efficient singlet oxygen generation could indeed be monitored by its characteristic NIR luminescence (1277 nm) for the three studied compounds^49^. Within experimental error, a generation efficiency close to unity was found for **T1** and **T2**, in line with the total quenching of their emission, while a significantly lower value of *ca* 0.78 was nonetheless monitored for **T3** (Supplementary Fig. 12). However, absorption spectra recorded on the latter before and after the quantification of singlet oxygen generation showed a *ca* 40% decrease of the absorption intensity at the irradiation wavelength. Thus, the singlet oxygen generation efficiency as reported herein for **T3** is probably significantly underestimated due to a chemical instability of the molecule and a propensity to photobleaching. Therefore, its real value may be closer to its mono-thionated counterparts **T1** and **T2**.

Barely discussed in most reported studies on thionated PSs, sensitivity of the series to photobleaching was thus investigated through in-time evolution of their singlet oxygen generation efficiency upon prolonged irradiation of 10 μM solution in CDCl_3,_ at their maximal absorption wavelength (Supplementary Fig. 13). In all three cases, a progressive, marked decrease of the singlet oxygen phosphorescence intensity was observed as irradiation time was increased. However, the evolution profile significantly differs. While in the cases of **T1** and **T2** the photobleaching process could be satisfactorily fitted to a first order kinetic model, with similar bleaching constants, **T3** exhibited a completely different evolution, with an initial fast disappearance rate, that could not be fitted to simple orders kinetic models (Supplementary Fig. 13). In order to investigate the potential photochemical transformation of **T1**-**T3**, we performed luminescence measurements under constant light irradiation on non-deoxygenated solutions (Supplementary Fig. 14). All compounds showed the emergence of an intense luminescent band with emission maximum centered at 502 nm, reminiscent of the reference **BTI** compound. Once cross tabulated with UV-visible experiments, it was found that for both **T1** and **T2**, the decrease of the long wavelength characteristic bands was concomitant to the increase of a 455 nm centered band, attributed to the back formation of the **BTI** compound, consistent with the kinetic following of singlet oxygen generation decay (Supplementary Fig. 14). In line with the latter, this evolution was characterized by the occurrence of an isosbestic point, indicative of a clean photoconversion process from both **T1** and **T2** to **BTI**. Regarding **T3**, UV-visible spectra indicated a more complex conversion profile, which also back-ups the aforementioned observation and appears consistent with the fact that the dithionated compound must first evolve into either one of the two possible monothionated derivatives, namely **T1** and **T2**. Indeed, **T1** and **T2** finally convert into the imide based **BTI**, giving raise, throughout the conversion process, to the formation of a complex and evolutive mixture of the four molecules (Supplementary Fig. 14). Conversely, solutions of thionated compounds were indeed found to be particularly stable when prepared and stored under inert atmosphere. Interestingly, prolonged UV irradiation of **BTI** in the presence of oxygen afforded its selective oxidation into a sulfone derivative (**BTI-SO**_**2**_^47^) as highlighted by UV-visible experiments (Supplementary Figs. 15, 16). Indeed, while the **BTI** characteristic band at ca 450 nm decreases in intensity, two isosbestic points at 282.5 and 418.5 nm were monitored together with the increasing of a band centered around 380 nm that fits with the optical patterns recorded on pure and chemically prepared **BTI-SO**_**2**_ (Supplementary Fig. 15b)^47^.

**Fig. 5:**
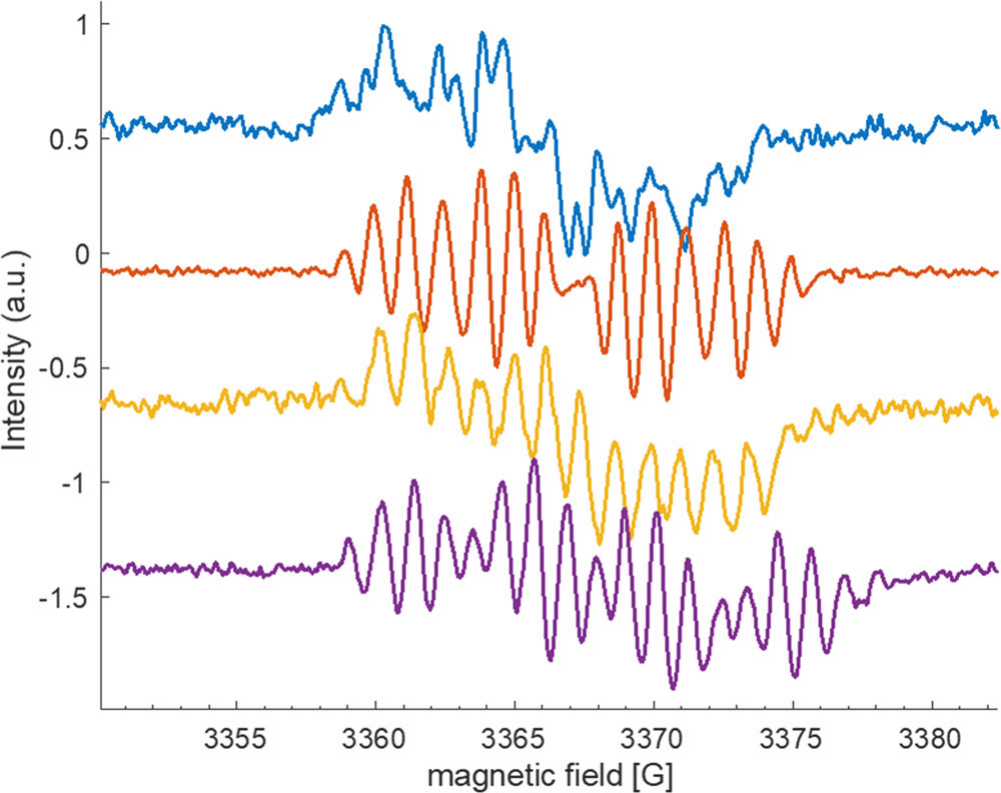
EPR spectroscopy. EPR spectra of **BTI** (blue line, g = 2.0033, aH (G) = 2.3; 2.2; 2.2; 1.85; 1.6; 1.6; 1.3; 0.5), **T1** (red line, g = 2.0044, aH (G) = 4.9; 2.88; 2.45; 1.44; 1.44; 1.08; 1.08; 1.08), **T2** (orange line, g = 2.0043, aH (G) = 2.7; 2.7; 1.3; 1.3; 1.1; 1.1; 2.2; 2.2) and **T3** (purple line, g = 2.0055, aH (G) = 4.4; 4.4; 4.4; 1.16; 1.16; 1.16; 1.16; 1.16) in DMSO.

ROS generation was also complementary investigated by electron paramagnetic resonance (EPR) experiments under light irradiation (530 nm in all cases), first using 2,2,6,6-tetramethyl-4-piperidone-N (TEMP) as a known specific singlet oxygen scavenger in chloroform^50^. A rapid increase of 2,2,6,6-tetramethyl-4-piperidone-N-oxyl radical (TEMPO) characteristic signal (g = 2.006, aN = 16.2 G, Supplementary Fig. 17)^50^ was monitored in time under continuous photoirradiation, witnessing intense singlet oxygen generation for the three photosensitizers. Conversely, when using 5,5-Dimethyl-1-pyrroline *N*-oxide (DMPO) as a radical scavenger (which can react with superoxide or other O- and C- centered radical species), no radical adducts could be observed even after long (ca 15 min) irradiation times. Similar experiments performed on aqueous suspensions of the photosensitizers did unfortunately not allow to observe such an evolution, which can be explained by an aggregation caused rapid quenching of the PS excited state, incompatible with triplet formation and thereby singlet oxygen generation (*vide infra*)^51^. In order to gain understanding on the ROS formation in environment of different polarities, characteristic of intracellular media^52^, studies in chloroform were then complemented by studies in DMSO. To our surprise, no singlet oxygen formation could be witnessed using TEMP as a scavenger, and the weak intensity radical signal initially observed (because of contamination of TEMP sample by traces of TEMPO) were rapidly consumed upon sample irradiation, indicating possible reduction of the latter through electron transfer from the PSs. Conversely, when DMPO was used as a scavenger, a modest growth of a DMPO-superoxide adduct signal^53^ (g = 2.0061, aN = 12.8 G, aH = 10.25 G, aH = 1.24 G, Supplementary Fig. 18) could be monitored at short irradiation times, which disappeared upon prolonged irradiation, with concomitant increase of a C-centered radical adduct signature. Indeed, when irradiation was performed on DMSO samples of PSs **T1–T3** devoid of scavenger, a similar persistent radical signature with broad envelope and complex, well resolved hyperfine splitting structure was observed upon prolonged sample irradiation (Fig. 5). This type of signal is characteristic of a delocalized radical on extended conjugated species, with hyperfine splitting resulting from interaction with the hydrogen substituents nuclear spin (see simulated spectra in Supplementary Fig. 19). Along with other abovementioned spin-trap experiments performed in DMSO, it suggests that oxygen sensitization in this solvent is mainly associated with photo-induced electron transfer from the PS to molecular oxygen, resulting in the formation of superoxide (O_2_^−•^) radical along with that of a PS centered radical cation. This hypothesis appears consistent with the low-lying in energy and reversible oxidation potential of the three molecules. Altogether, these results indicate that the potential photo-cytotoxicity of the studied compounds *(vide infra)* may be driven by an interplay of type I and type II PDT mechanisms.

This latter observation led us to undertake EPR investigation on the parent **BTI** compound as well: while our spectroscopic investigations pointed out a high luminescence efficiency and a singlet oxygen generation below our detection limit, a priori precluding its use as a singlet oxygen photosensitizer, no information was available regarding its potential propensity towards superoxide generation through photoinduced electron transfer. A similar set of experiments as previously described for **T1**–**T3** were thus carried out with **BTI**, using a 455 nm LED as an irradiation source. To our surprise, experiments carried out in chloroform using TEMP as a scavenger showed some formation of TEMPO characteristic of singlet oxygen generation (Supplementary Fig. 17c), albeit with a much lower kinetic than that monitored for **T1** and **T2** (Supplementary Fig. S17d). **T3** has been excluded from this study, because of its lack of stability upon prolonged irradiation. Conversely, experiments carried out with DMPO in DMSO solvent showed a rapid increase of a superoxide adduct signature (Supplementary Fig. S18). In the absence of spin-trap, a similar complex persistent radical signature as monitored for **T1**–**T3** was recorded (Fig. 5 and Supplementary Fig. 19), again indicative of the occurrence of a photo-induced electron transfer from the PS to molecular oxygen. These data led us to reconsider a possible use of **BTI** as a PDT photosensitizer.

### Self-assembly properties and intracellular ROS production

**Fig. 6:**
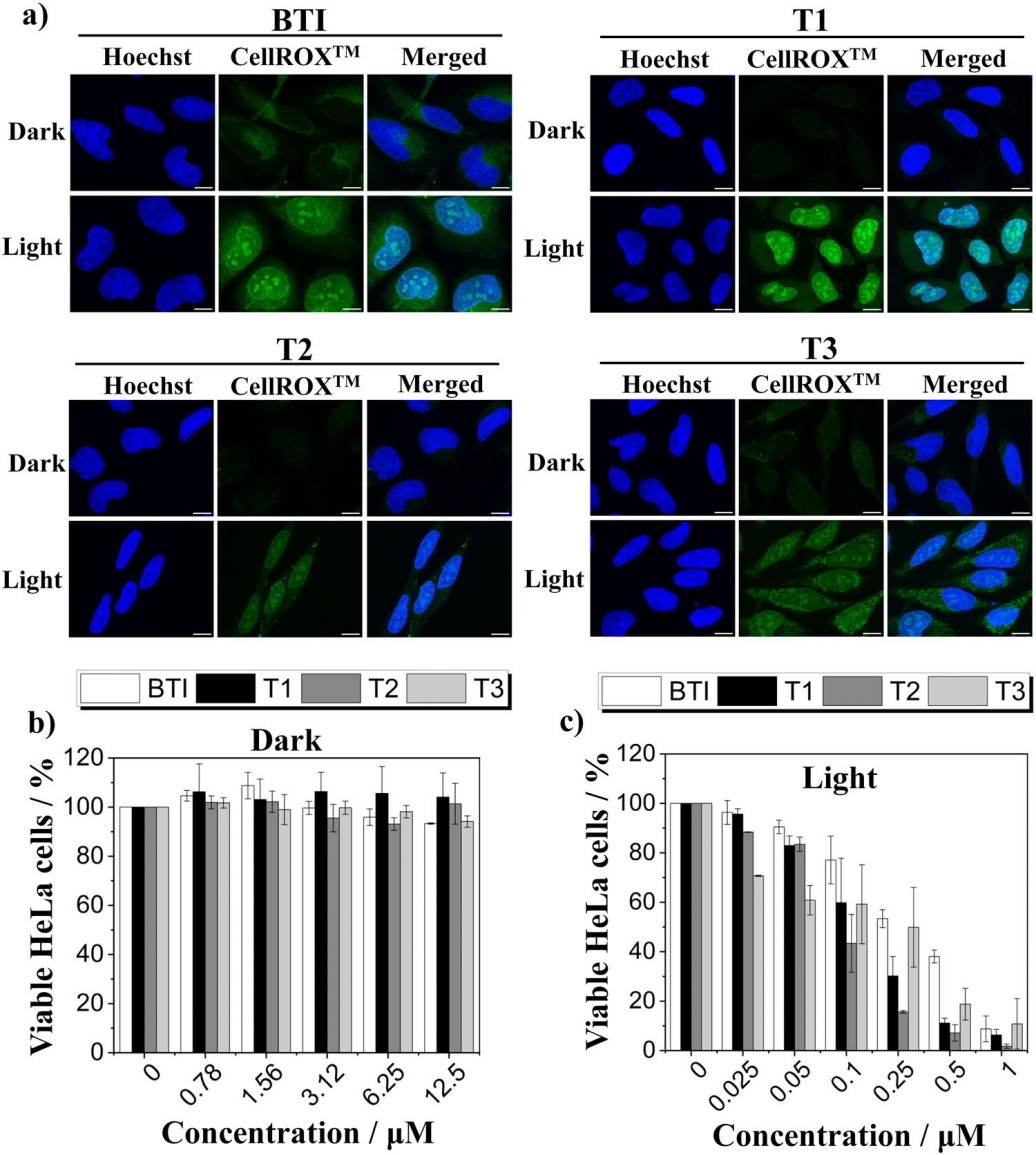
Intracellular ROS production and phototherapeutic activity. **a** HeLa cells were treated with **BTI**, **T1**, **T2**, or **T3** (500 nM) and incubated at 37 °C for 24 h. Afterwards, the cells were incubated with CellROX^TM^ green reagent (5 µM, green signal) for 30 min at 37 °C, and where indicated, blue light generated by a LED light cube (excitation = 470/22 nm, 23 mW cm^−2^) was applied to the cells for 10 min. Hoechst 33342 (500 nM, blue signal) was added to the cells for 30 min at 37 °C before imaging. Hoechst 33342: λ_exc_/λ_em_: 405/430–460 nm and CellROX^TM^: λ_exc_/λ_em_: 490/510–670 nm. Scale bar 10 µm. Cytotoxicity studies of **BTI**, **T1**, **T2**, and **T3** on HeLa cells in the absence (**b**) or presence (**c**) of blue light (excitation = 470/22 nm, 23 mW cm^−2^) irradiated for 6 min. *n* = 3 independent experiments. Mean ± SD is indicated.

In aqueous solution, **BTI**, **T1**, **T2**, and **T3** showed the propensity to form nanoaggregates (**BTI** = 316 nm, **T1** = 100 nm, **T2** = 172 nm, **T3** = 151 nm) in line with previously published data reporting on the use of thionaphthalimides^37^ or thione-derived perylene diimides^41^ as activated-self-assembled PSs (Supplementary Fig. 20). It is important to note that the propensity of self-assembled dyes to disaggregate into monomeric units upon binding to biological targets in cell culture conditions has been used in both biosensing^54–57^ and therapeutic applications^37,41,58^. This would be a particularly important expected feature in the present study, as we already mentioned that no singlet-oxygen or other ROS generation could be monitored in aqueous media, while singlet oxygen and superoxide generation could be attested in chloroform and DMSO, respectively. To verify the assembly/disassembly character of the compounds, we used bovine serum albumin (BSA) as a targeted protein. Indeed, the addition of BSA to **BTI**, **T1**, **T2**, and **T3** caused the disruption of the aggregates (**BTI**-BSA = 15 nm, **T1**-BSA = 1 nm, **T2**-BSA = 19 nm, **T3**-BSA = 14 nm) (Supplementary Fig. 20). Next, we investigated the ability of **T1**–**T3** to photo-generate ROS in human cervical epithelioid carcinoma (HeLa) cells. Following our EPR experiments which underlined its good ability to induce superoxide formation and more generally electron transfer upon photoexcitation, **BTI** was also included in this set of experiments. CellROX™ green reagent was used as a fluorogenic probe for detecting oxidative stress in the nucleus of live cells. This dye is weakly fluorescent in a reduced state while displaying bright green fluorescence upon oxidation by ROS. As expected, ROS generation was not observed when the cells were incubated with the compounds under dark condition (Fig. 6a). Conversely, upon blue light exposure, a bright green fluorescence signal was observed demonstrating the ability of the compounds to photo-generate ROS (Fig. 6a). These results prompted us to study their potential use as phototherapeutic agents. HeLa cells treated with different concentrations of **BTI**, **T1**, **T2**, and **T3** for 48 h in the dark showed negligible cytotoxic effects (Fig. 6b). Conversely, under blue-LED illumination, **BTI**, **T1**, **T2**, and **T3** induced cancer cell death in a concentration-dependent manner (Fig. 6c).

The phototherapeutic efficacy derived by calculating the half maximal inhibitory concentration (IC_50_) was found to follow the order: **T2** (IC_50_ = 96 ± 9 nM) > **T3** (IC_50_ = 136 ± 54 nM) > **T1** (IC_50_ = 138 ± 10 nM) > **BTI** (IC_50_ = 279 ± 11 nM). These experiments clearly demonstrate the efficiency of thionated compounds, particularly **T2**, to induced cancer cell death. Furthermore, the bathochromic shift resulting from the successive thionations of **T1**–**T3** can be exploited for phototherapeutic purposes. Indeed, **T2** exhibited, under green-LED illumination, a photoinduced cancer cell death similar to blue light irradiation (Supplementary Fig. 21). Similarly, **T3** showed potent photo-driven cell death upon orange-LED illumination (Supplementary Fig. 22). Overall, these data show that sulfur-substitution provides PSs with enhanced phototoxic effects and tunable optical properties suited for PDT.

**Fig. 7:**
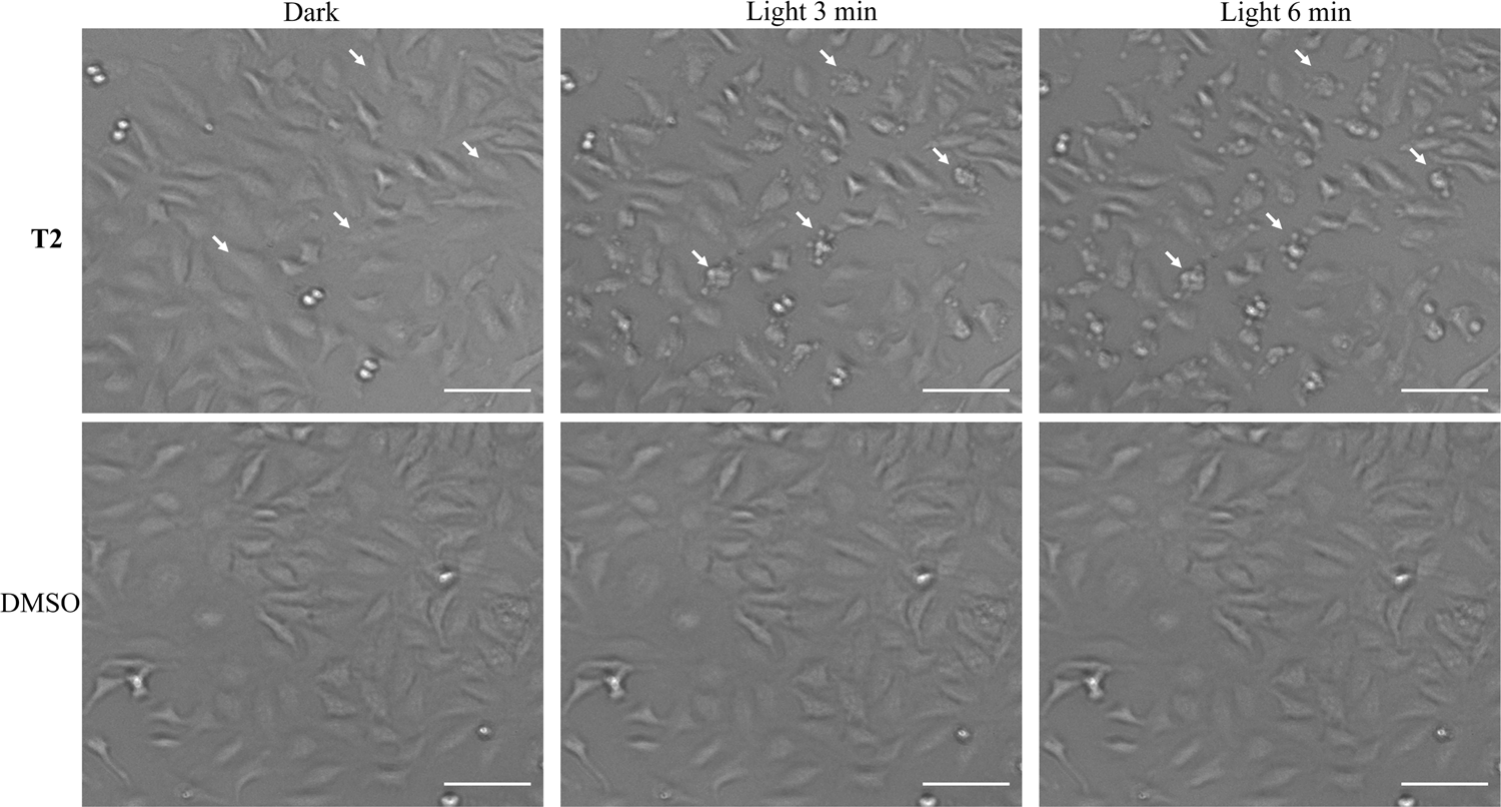
Light-induced cancer cell death. Time-lapse experiments of live HeLa cells. Upper panel: light-induced morphological changes of **T2**-treated (1 μM) cells after 0, 3, and 6 min of blue light irradiation. Lower panel: Control experiments performed with DMSO-treated HeLa cells. White arrows indicate different cells and how their morphology changes during the time-lapse. Scale bar = 100 μm.

Finally, real-time monitoring of morphological changes in living HeLa cells subjected to **T2** treatment and blue or green light irradiation unraveled dramatic alterations of the cellular architecture that literally resulted in the “explosion” of the cancer cells into small apoptotic bodies, characteristic of a very efficient PDT process (Fig. 7 and Supplementary Fig. 23)^59^.

## Conclusions

In summary, we have reported on a molecular design approach that provides easy access to simple, efficient, and synthetically accessible heavy-atom-free PSs through the sequential thionation of the benzothioxanthene imide chromophore. Experimental and computational studies shed light on the role exerted by the sulfur atoms to impact both the opto-electronic properties and singlet oxygen or superoxide generation ability of the PSs. By complementary spectroscopy techniques, we showed that the photophysical properties of the three PSs drastically depend on solvents polarity. While quantitative singlet oxygen generation was monitored in low polarity solvent (chloroform) for **T1, T2**, and **T3**, generation of superoxide was monitored in DMSO which was found particularly strong for **BTI** leading us to postulate a photoredox electron transfer mechanism in this high polarity solvent. This was further supported by the formation of a persistent PS´s centered cation radical on **T1, T2**, **T3**, and **BTI**. This rather unexpected behavior suggests that multiple activation pathways (of type II, with singlet oxygen and/or superoxide radicals, and type I, with direct electron transfer) may participate to the cellular photo-cytotoxicity of this series of molecules depending on the targeted organelle polarity. The potential use of the compounds as PSs for PDT was therefore confirmed by cellular experiments. To conclude, this study provides a new platform for structural control and functional design of novel phototherapeutics for PDT.

## Methods

### General information

All reagents and chemicals from commercial sources were used without further purification. Solvents were dried and purified using standard techniques. Flash chromatography was performed with analytical-grade solvents using Aldrich silica gel (technical grade, pore size 60Å, 230–400 mesh particle size). Flexible plates ALUGRAM® Xtra SIL G UV254 from MACHEREY-NAGEL were used for TLC. Compounds were detected by UV irradiation (Bioblock Scientific). NMR spectra were recorded with a Bruker AVANCE III 300 (^1^H, 300 MHz and ^13^C, 75 MHz) or a Bruker AVANCE DRX500 (^1^H, 500 MHz; ^13^C, 125 MHz). Chemical shifts are given in ppm relative to TMS and coupling constants J in Hz. Matrix Assisted Laser Desorption/Ionization was performed on MALDI-TOF MS BIFLEX III Bruker Daltonics spectrometer using DCTB+ as matrix. High resolution mass spectrometry (HRMS) was performed with a JEOL JMS-700 B/E.

### Synthesis

Lawesson’s reagent (433 mg, 1.07 mmol) was added to a solution of **BTI** (200 mg, 535 µmol) solubilized in o-Dichlorobenzene (10 mL). After heating at 160 °C for 1h30 under argon atmosphere, the solution was cooled to room temperature and the solvent evaporated under vacuum. The crude was then washed with water and extracted with chloroform, dried with MgSO4 and concentrated under vacuum before being purified by column chromatography on silica gel using toluene as eluent.

**T1** (13%): ^1^H NMR (300 MHz, Chloroform-d): δ 8.95 (dd, J = 8.5, 1.3 Hz, 1H), 8.58 (dd, J = 8.3, 1.3 Hz, 1H), 8.26–8.14 (m, 2H), 7.49–7.33 (m, 4H), 6.36 (m, 1H), 2.29 (m, 2H), 2.03 (m, 2H), 0.93 (t, J = 7.5 Hz 6H). ^13^C NMR (126 MHz, Chloroform-d): δ 196.7, 161.1, 140.6, 138.0, 136.6, 133.2, 131.8, 130.2, 128.7, 128.1, 127.9, 126.6, 126.4, 125.3, 124.7, 122.4, 121.1, 119.8, 65.3, 25.3, 11.4. HRMS (FAB-neg): calc. for C_23_H_19_NOS_2_: 389.0908, found 389.0908.

**T2** (19%): ^1^H NMR (300 MHz, Chloroform-d): δ 9.10 (d, J = 8.6 Hz, 1H), 8.38 (d, J = 8.0 Hz, 1H), 8.23–8.15 (m, 1H), 8.11 (d, J = 8.6 Hz, 1H), 7.48 (d, J = 8.1 Hz, 1H), 7.47–7.32 (m, 3H), 6.33 (m, 1H), 2.30 (m, 2H), 2.02 (m, 2H), 0.93 (t, J = 7.5 Hz, 6H). ^13^C NMR (126 MHz, Chloroform-d): δ 196.1, 161.6, 140.8, 139.8, 136.1, 132.1, 131.3, 130.2, 128.8, 128.2, 127.9, 127.2, 126.5, 126.3, 125.5, 121.0, 119.8, 119.3, 65.4, 25.4, 11.4. HRMS (FAB-neg): calc. for C_23_H_19_NOS_2_: 389.0908, found 389.0909.

**T3** (16%): ^1^H NMR (300 MHz, Chloroform-d): δ 8.99 (bs, 1H), 8.82 (bs, 1H), 8.23–8.12 (m, 1H), 8.08 (d, J = 8.7 Hz, 1H), 7.49–7.34 (m, 4H), 6.75 (m, 1H), 2.61 (m, 2H), 2.20 (m, 2H), 0.93 (t, J = 7.5 Hz, 6H). ^13^C NMR (126 MHz, Chloroform-d): δ 140.6, 138.6, 138.2, 136.2, 130.2, 128.1, 126.5, 126.4, 125.2, 125.0, 121.7, 120.4, 70.5, 24.9, 11.36. HRMS (FAB-neg): calc. for C_23_H_19_NS_3_: 405.0680, found 405.0694.

### X-ray diffraction data

X-ray single-crystal diffraction data were collected on a Rigaku Oxford Diffraction SuperNova diffractometer equipped with Atlas CCD detector and micro-focus Cu-K_α_ radiation (λ = 1.54184Å) for **BTI** and molecule **T3**. For molecules **T1** and **T2**, crystal data were collected on a BRUKER KappaCCD diffractometer, equipped with a graphite monochromator utilizing MoKα radiation (λ = 0.71073Å). The structures were solved by direct methods and refined on F^2^ by full matrix least-squares techniques using SHELX programs (G. M. Sheldrick 2014–2016, SHELXT 2014/5 and SHELXL 2016/4). All non-H atoms were refined anisotropically and multiscan empirical absorption was corrected using CrysAlisPro program (CrysAlisPro, Agilent Technologies, V1.171.40.45, 2019) for **BTI** and molecule **T3** and using SADABS program (Sheldrick, Bruker, 2008) for molecules **T1** and **T2**. The H atoms were included in the calculation without refinement. CCDC 2083265 (**BTI**), 2116980 (**T1**), 2116981 (**T2**) and 2116982 (**T3**) contains the supplementary crystallographic data for this paper.

### Cyclic voltammetry

Cyclic voltammetry was performed using a Biologic SP-150 potentiostat with positive feedback compensation in 0.10 M Bu_4_NPF_6_/CH_2_Cl_2_ (HPLC grade). Experiments were carried out in a one-compartment cell equipped with a platinum working electrode (2 mm of diameter) and a platinum wire counter electrode. A silver wire immersed in 0.10 M Bu_4_NPF_6_/CH_2_Cl_2_ was used as pseudo-reference electrode and checked against the ferrocene/ferrocenium couple (Fc/Fc^+^) before and after each experiment. The potentials were then expressed vs Fc/Fc^+^.

### Optical measurements and singlet oxygen quantum yield

Absorption spectra were recorded on a JASCO V-650 spectrophotometer in diluted solution (ca. 10^–5^ or 10^–6^ M), using spectrophotometric grade solvents. Emission spectra were measured using Horiba-Jobin–Yvon Fluorolog-3 fluorimeter, equipped with a standard R928 detector for the visible and a liquid nitrogen cooled, solid Indium/Gallium/Arsenic detector (850–1600 nm) for NIR measurements of the singlet oxygen phosphorescence signal. Solutions were excited by unpolarized light from a 450 W xenon continuous wave (CW) lamp and detected at an angle of 90° for measurements of dilute solutions (10 mm quartz cuvette). Spectra were corrected for both excitation source light-intensity variation (excitation being performed at the maximal wavelength for each compound; **T1**, **T2**: λ_ex_ = 515 nm; **T3**: λ_ex_ = 585 nm) and photodetector spectral responses (dark offset). Singlet oxygen quantum yield (φ_Δ_) measurements were achieved using a relative methodology based on the comparison of singlet oxygen (^1^O_2_) phosphorescence intensity of diluted CDCl_3_ solutions (OD < 0.1) of studied samples against that of Phenalenone in the same solvent (φ_Δ_ = 0.95) used here as a reference. Singlet oxygen luminescence quantum yields were calculated using the following Eq. 1:

1 $${{{{{{\rm{\varphi }}}}}}}_{\Delta {{{{{\rm{x}}}}}}}/{{{{{{\rm{\varphi }}}}}}}_{\Delta {{{{{\rm{r}}}}}}}=[{{A}}_{{{{{{\rm{r}}}}}}}({{{{{{\rm{\lambda }}}}}}}_{{{{{{\rm{r}}}}}}})/{{A}}_{{{{{{\rm{x}}}}}}}({{{{{{\rm{\lambda }}}}}}}_{{{{{{\rm{x}}}}}}})][{{D}}_{{{{{{\rm{cx}}}}}}}/{{D}}_{{{{{{\rm{cr}}}}}}}]$$

where A(λ) is the absorbance (or optical density) at the excitation wavelength and *D*_c_ the corrected integrated luminescence intensity. The subscripts r and x stand for reference and sample, respectively. Excitation slits were set at 5 nm, emission slits at 15 nm, integration time was 1 s. The reported results are the average of 3–4 independent measurements at various absorbances (comprised between 0.01 and 0.1) for both sample and reference. The plot of the integrated luminescence intensity *vs*. absorbance gives straight line with excellent correlation coefficients and the slope S can be determined for both sample and reference, allowing determination of the sample ^1^O_2_ generation efficiency.

Photobleaching experiments were performed using the same setup, using the kinetic mode of the spectrofluorometer and a detection wavelength λ_em_ = 1277 nm.

### EPR spectroscopy

EPR assays were all carried out on a Bruker E500 spectrometer operating at X-band (9.44 GHz), standard cavity, with 100 KHz modulation frequency. Experiments were all performed at room temperature. The instrument settings were as follows: microwave power in the 2–69 mW range; modulation amplitude of 0.5–1 G. The irradiation using a Thorlab LED 530 nm or Thorlab LED 455 nm source was directly performed into the EPR cavity during spectra recording. Hyperfine coupling constants values were obtained by simulating experimental spectra with Easyspin (Matlab toolbox) and compared with the literature in the case of the spin trap adducts^60–62^. Spin-trapping agents TEMP (Aldrich) and DMPO (TCI) were used immediately after opening, and samples were prepared in air-open capillary tubes. DMSO and chloroform solvents of spectroscopic grade were used. In all cases, PS concentration was 0.1 mM, while concentration of TEMP or DMPO scavenger, when present, was set as 5 mM.

### Dynamic light scattering (DLS)

Dynamic light scattering (DLS) were recorded with a Malvern Instruments Zetasizer nano series instrument. Aqueous suspensions (0.5 μM) were prepared by nanoprecipitation of a concentrated (2.5 mM) solution of **BTI**, **T1**, **T2**, and **T3** in DMSO. Bovine serum albumin (Sigma-Aldrich, Ref No: A9647) was prepared in water at a final concentration of 1 mM. Samples were left to equilibrate for 1 h before DLS measurements.

### Photoinduced intracellular ROS generation

For all biological experiments, HeLa cells were cultured at 37 °C in 5% CO_2_ in DMEM medium supplemented with penicillin-streptomycin (1×), and 10% fetal bovine serum. 15 × 10^4^ HeLa cells were seeded the day before treatment on glass-bottomed microwell dishes (MatTek Corp.). HeLa cells were treated with **BTI**, **T1**, **T2**, or **T3** (stock solutions: 2.5 mM in DMSO) dissolved in complete medium at 1 µM and incubated at 37 °C in 5% CO_2_ for 24 h. CellROX^TM^ green reagent (Invitrogen, Ref No: C10444) (5 µM) was added to the cells for 30 min at 37 °C in 5% CO_2_. When required, the cells were photo-irradiated by using an EVOS® FL cell imaging system equipped with an adjustable-intensity LED cube (excitation = 470/22 nm) operating at 23 mW cm^−2^ for 10 min. Then, cells were washed twice with 1× PBS. Finally, the cells were kept into live cell imaging solution (Molecular Probes^TM^, Ref No: A14291DJ) and imaged by using a Leica SP8 FALCON confocal microscope. Maximum intensity projection of Z-stack images was used for data presentation. All data were processed by using ImageJ software.

### Photo-cytotoxicity on HeLa cells

5 × 10^3^ cells/well were seeded in complete medium on 96 well-plates 24 h before compounds treatment. **BTI**, **T1**, **T2**, and **T3** were dissolved in complete medium at the indicated concentrations (DMSO reached the max value = 0.5 % v/v) and added to cells for 24 h at 37 °C in 5% CO_2_. When required, the cells were photo-irradiated by using a EVOS® FL cell imaging system equipped with an adjustable-intensity LED cube (Blue light: excitation = 470/22 nm) operating at 23 mW cm^−2^ for 6 min. **T2**- or **T3**-treated HeLa cells were also subjected to green or orange light irradiation, respectively. Adjustable-intensity LED cubes (Green light: excitation = 542/20 nm or Orange light: excitation = 585/29 nm) operating at 10 and 15.5 mW cm^−2^ for 6 or 14 min, respectively, were used to deliver the light. Then, the cells were incubated for additional 24 h at 37 °C in 5% CO_2_. At 48 h after compounds treatment, PrestoBlue (Invitrogen, Ref No: A13261) was added to each well and the cells were incubated at 37 °C in 5% CO_2_ for 3 additional h. Cell viability was measured by recording the fluorescence signal of PrestoBlue (λ_exc_/λ_em_: 560/590 nm) using a Synergy H4 microplate reader (Biotek). Each experiment was performed in duplicates/triplicates from distinct samples.

### Light-induced morphological changes on T2-treated HeLa cells

5 × 10^3^ cells/well were seeded in complete medium on 96 well-plates 24 h before treatment with **T2**. **T2** (1 μM) or an equivalent amount of DMSO, was dissolved in complete medium and added to cells for additional 24 h at 37 °C in 5% CO_2_. Then, the cells were photo-irradiated by using an EVOS® FL cell imaging system equipped with adjustable-intensity LED cubes (Blue light: excitation = 470/22 nm operating at 27 mW cm^−2^ or Green light: excitation = 542/20 nm operating at 10 mW cm^−2^) for 6 min.

### Computational details

The Gaussian16 code^47^ was used to optimize the geometries at the ground and excited states along with the global hybrid functional PBE0^63^. This functional was chosen because of its accuracy to reproduce spectroscopic properties of **BTI**-based molecules. Structural optimizations and subsequent frequency calculations for both the ground and excited states were performed using an all electron Pople triple zeta basis set with one polarization function on all atoms and one diffuse function of heavier atoms, known as 6–311 + G(d,p), for H, C, N, O and S atoms^64^. Bulk solvent effects were included using the Polarizable Continuum Model (PCM) of Tomasi and co-workers^65^. Default radii (from the UFF, scaled by 1.1) were used. Excited state geometry was obtained by minimizing the forces of the S_1_ state computed at the TD-DFT level by considering the 3 first excited states. The Dalton^66^ program was used to compute the SOC between the three first triplet states (namely T_1_, T_2_, and T_3_) and the S_1_ state at the S_1_ optimized geometry using the quadric-response TD-DFT at the B3LYP/PCM level with the aug-cc-pVDZ basis set adapted for the Douglas–Kroll calculations^67^. The spin-orbit coupling was computed using the Douglas–Kroll Hamiltonian along with the spin-orbit mean field approach^68^.

### Reporting summary

Further information on research design is available in the Nature Research Reporting Summary linked to this article.

## Supplementary information

Supplementary Information

Reporting Summary

## Data Availability

The data that support this study are present in the manuscript and supplementary information and are available from the corresponding author upon request.
