# Supplementary material for: Site-selected thionated benzothioxanthene chromophores as heavy-atom-free small-molecule photosensitizers for photodynamic therapy
Source: Commun Chem. 2022 Oct 31;5:142. doi: 10.1038/s42004-022-00752-x (PMC9814739; doi:10.1038/s42004-022-00752-x)
Supplement: Supplementary file 1 — Supplementary Information [file 42004_2022_752_MOESM1_ESM.pdf]

Supplementary Information

Site-Selected Thionated Benzothioxanthene Chromophores as Heavy-Atom-Free Small-Molecule Photosensitizers for Photodynamic Therapy

Marco Deiana[1#], Pierre Josse[2#], Clément Dalinot[2], Artem Osmolovskyi[2], Pablo Simón Marqués[2], José María Andrés Castán[2], Laura Abad Galán[3], Magali Allain[2], Lhoussain Khrouz[3], Olivier Maury[3], Tangui Le Bahers[3], Philippe Blanchard[2], Sylvie Dabos-Seignon[2], Cyrille Monnereau[3*], Nasim Sabouri[1*] and Clément Cabanetos[2,4*]

[1] Department of Medical Biochemistry and Biophysics, Umeå University, SE-901 87 Umeå, Sweden. E-mail: nasim.sabouri@umu.se

[2] Univ Angers, CNRS, MOLTECH-ANJOU, SFR MATRIX, F-49000 Angers, France. E-mail: clement.cabanetos@cnrs.fr

[3] Univ Lyon, ENS de Lyon, CNRS UMR 5182, Université Claude Bernard Lyon 1, F-69342 Lyon, France. E-mail: cyrille.monnereau@ens-lyon.fr

[4] IRL CNRS 2002, 2BFUEL, CNRS -Yonsei University, Seoul, South Korea

[#] These authors contributed equally

Table of contents

NMR Spectra

HRMS Spectra

Comparative ^{1}H NMR spectra

X-ray diffraction data

Singlet oxygen quantum yield

Photostability studies

Photoconversion of T1, T2, T3 and BTI

EPR spectroscopy

Dynamic light scattering

Phototoxic effects

Green light-induced morphological changes of HeLa cells

NMR Spectra

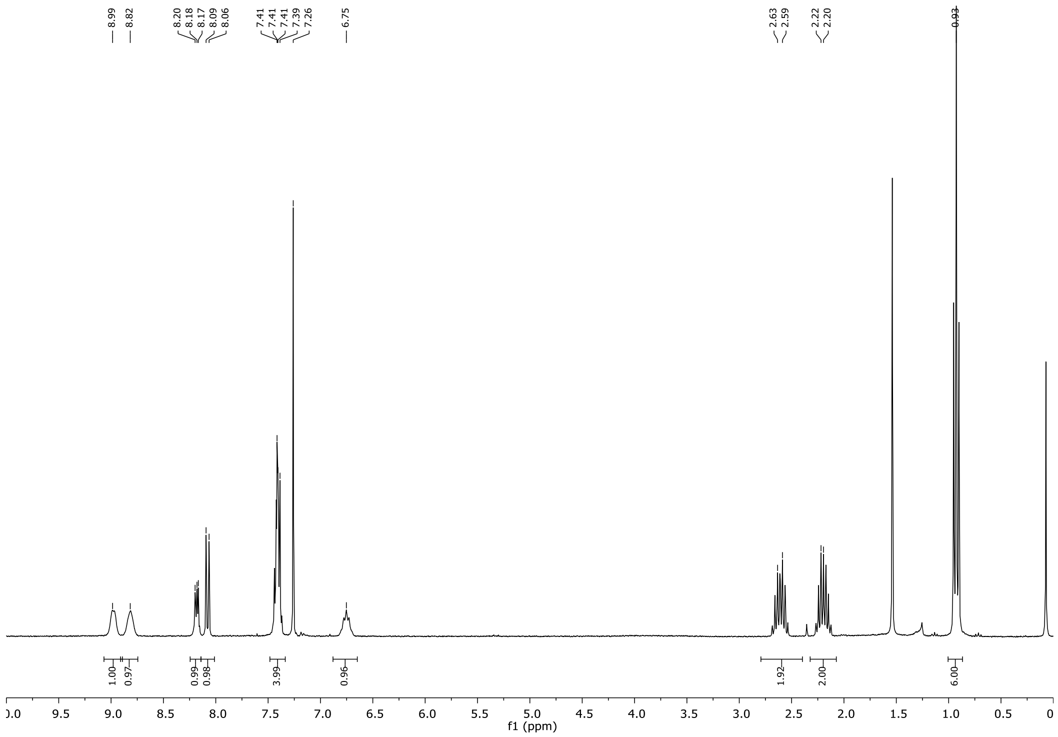

Supplementary Figure 1. ^{1}H NMR **T3**.

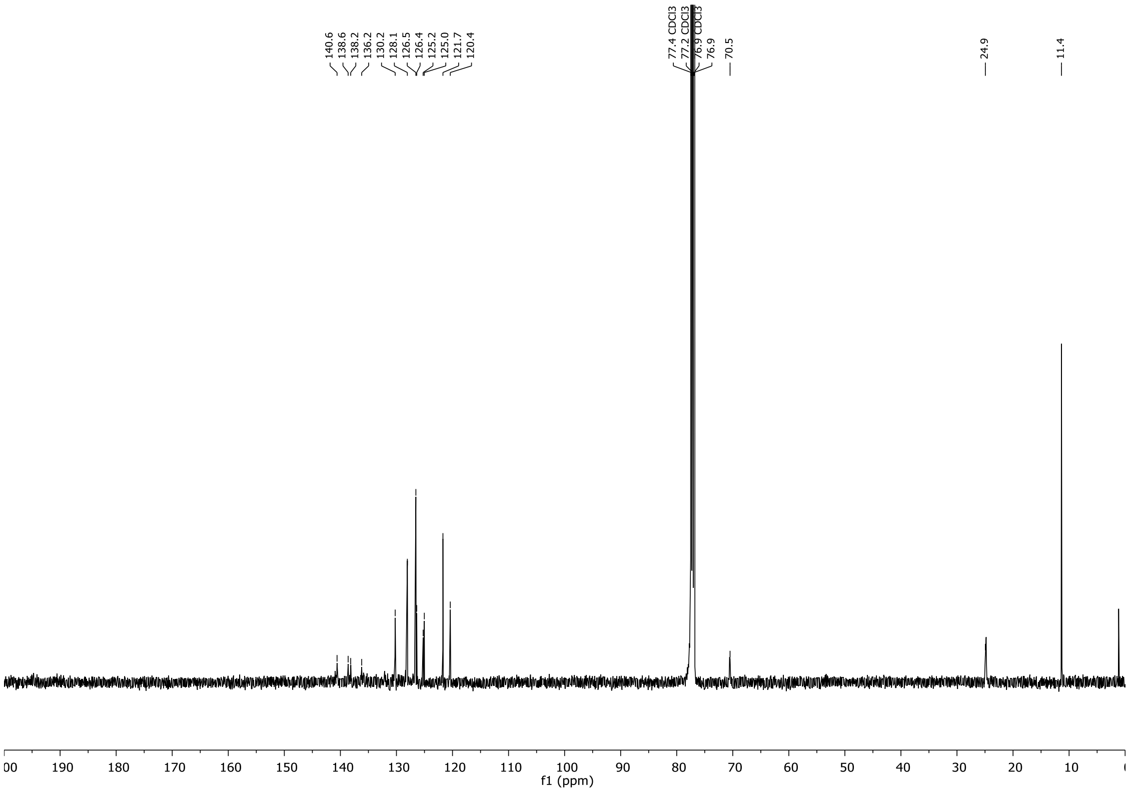

Supplementary Figure 2. ^{13}C NMR **T3**.

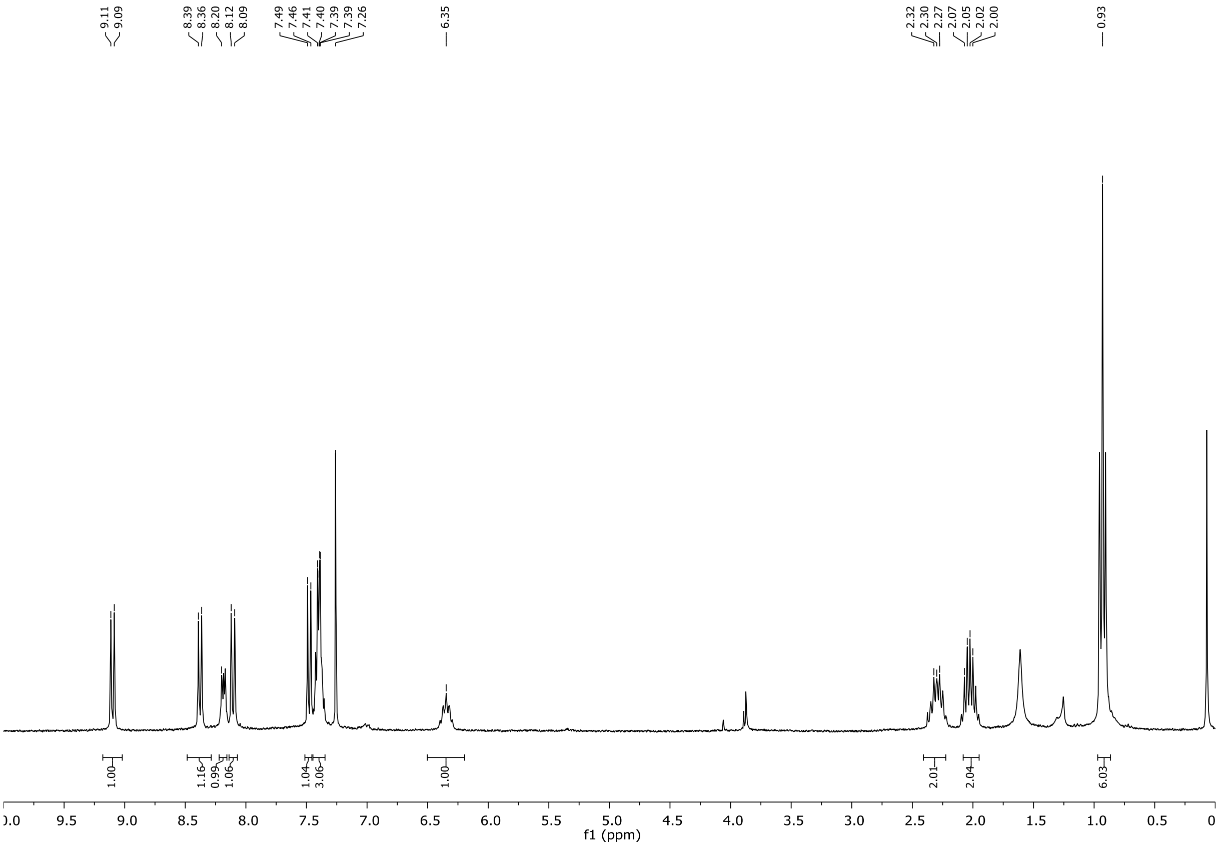

Supplementary Figure 3. ^{1}H NMR **T2**.

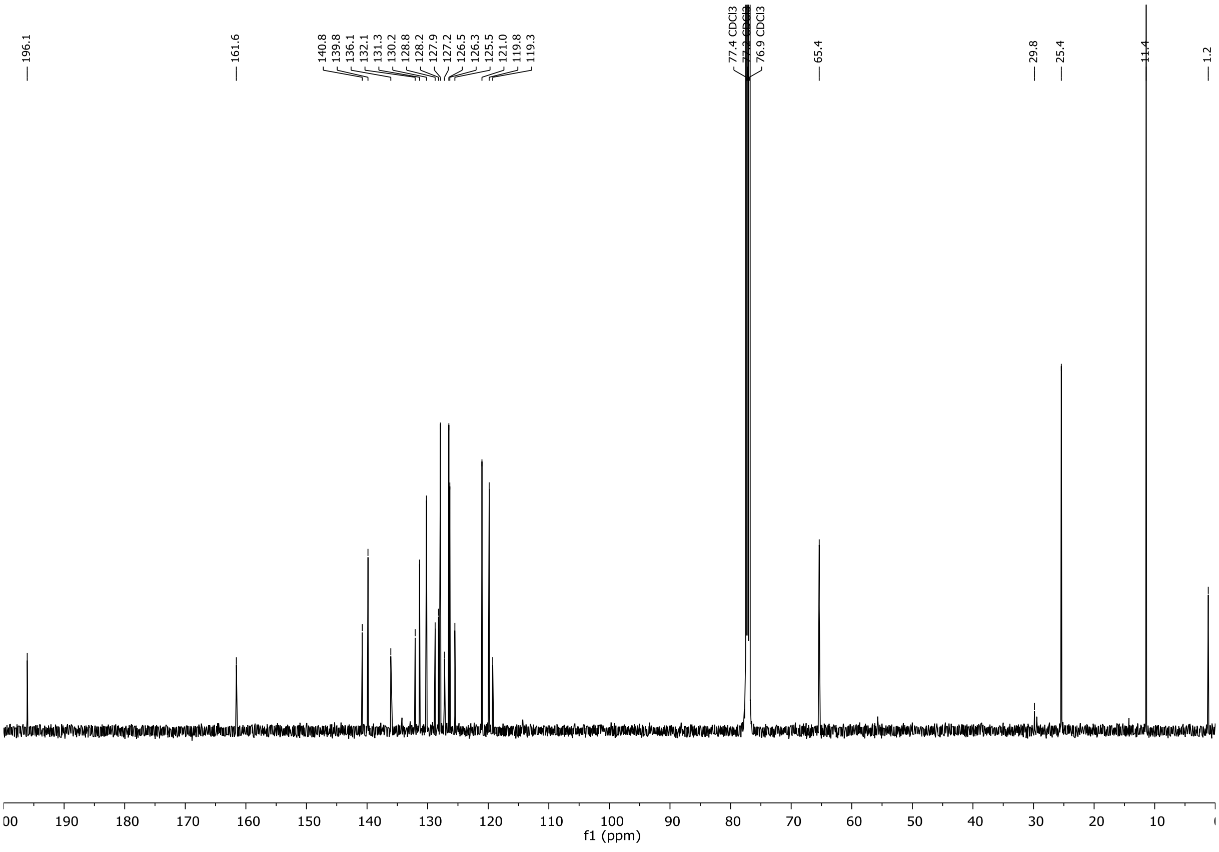

Supplementary Figure 4. ^{13}C NMR **T2**.

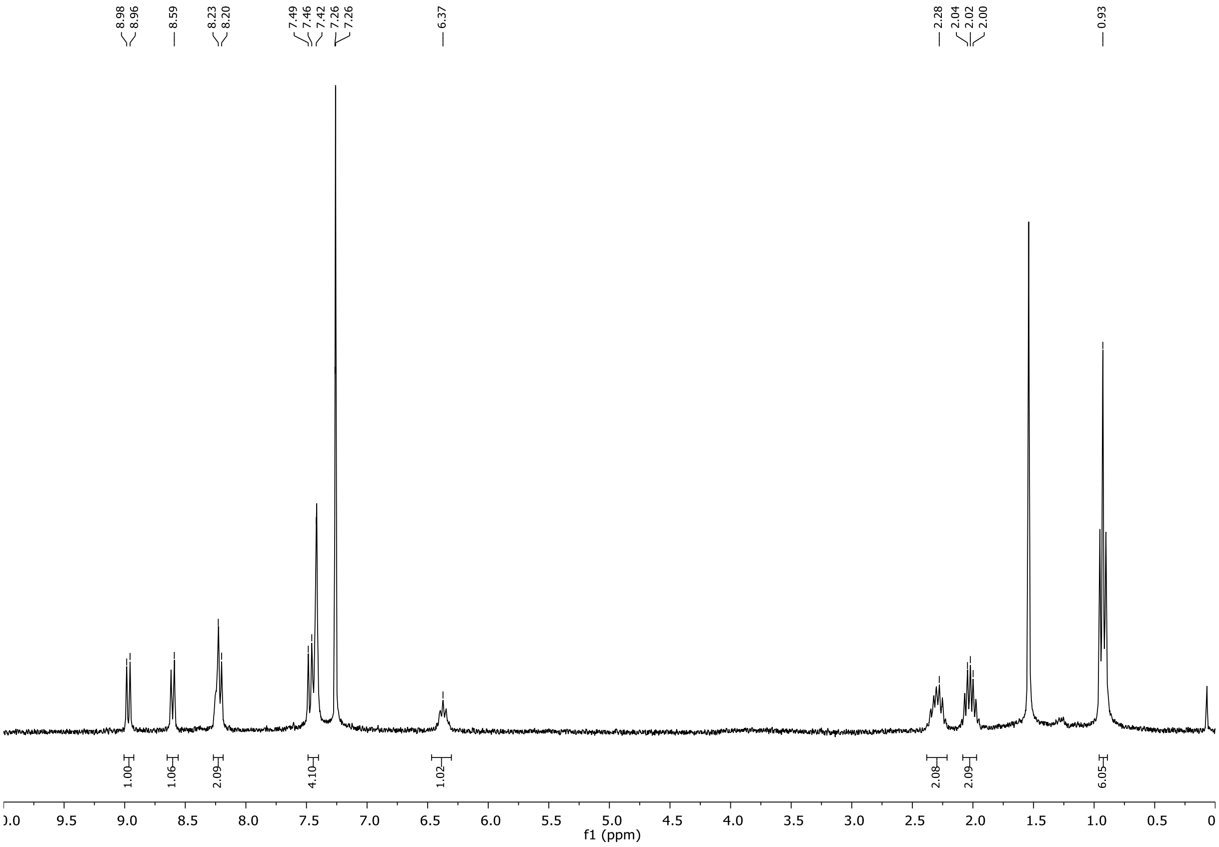

Supplementary Figure 5. ^{1}H NMR **T1**.

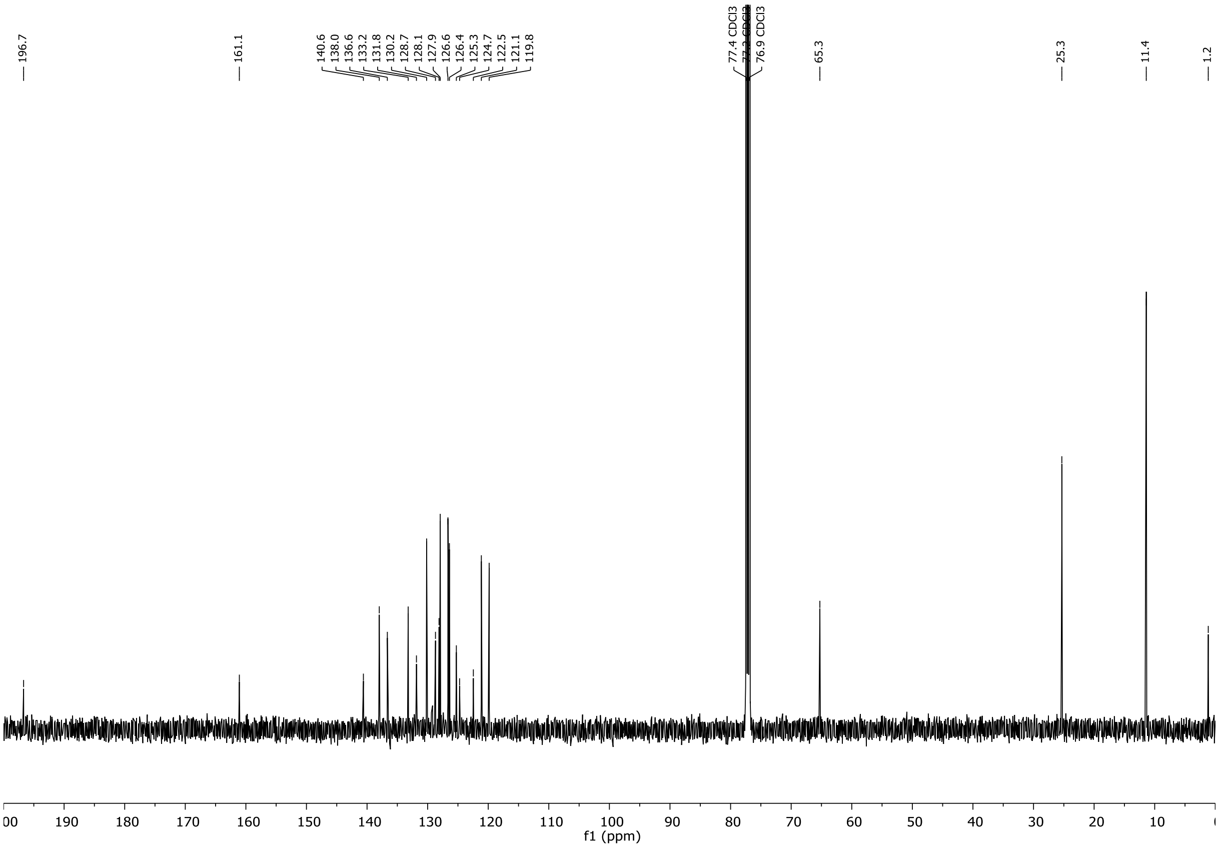

Supplementary Figure 6. ^{13}C NMR **T1**.

HRMS Spectra

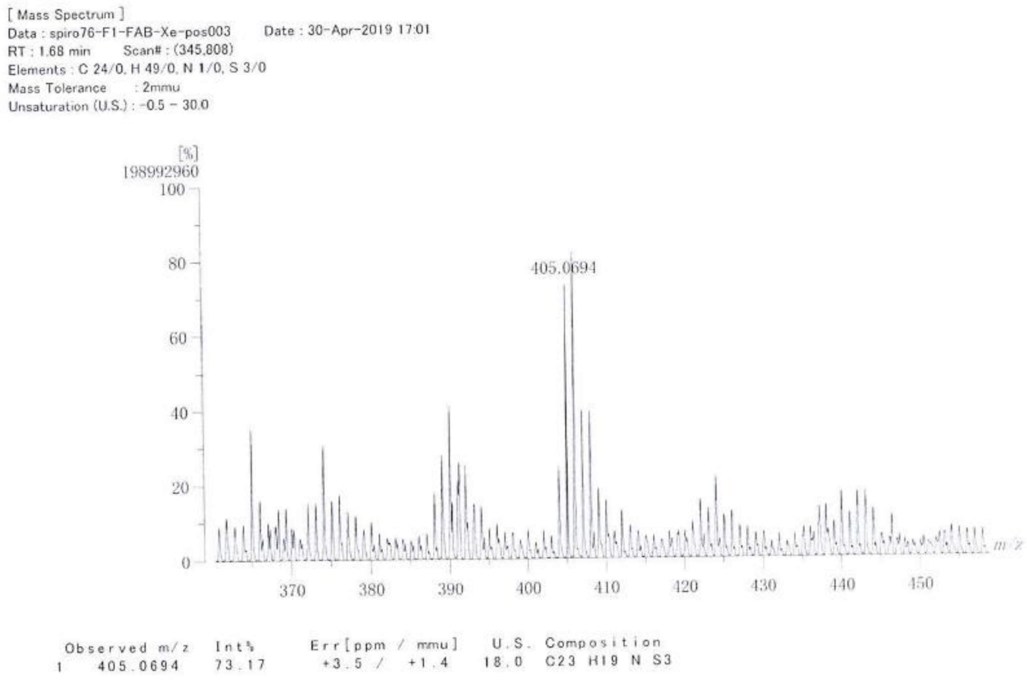

Supplementary Figure 7. HRMS of **T3**.

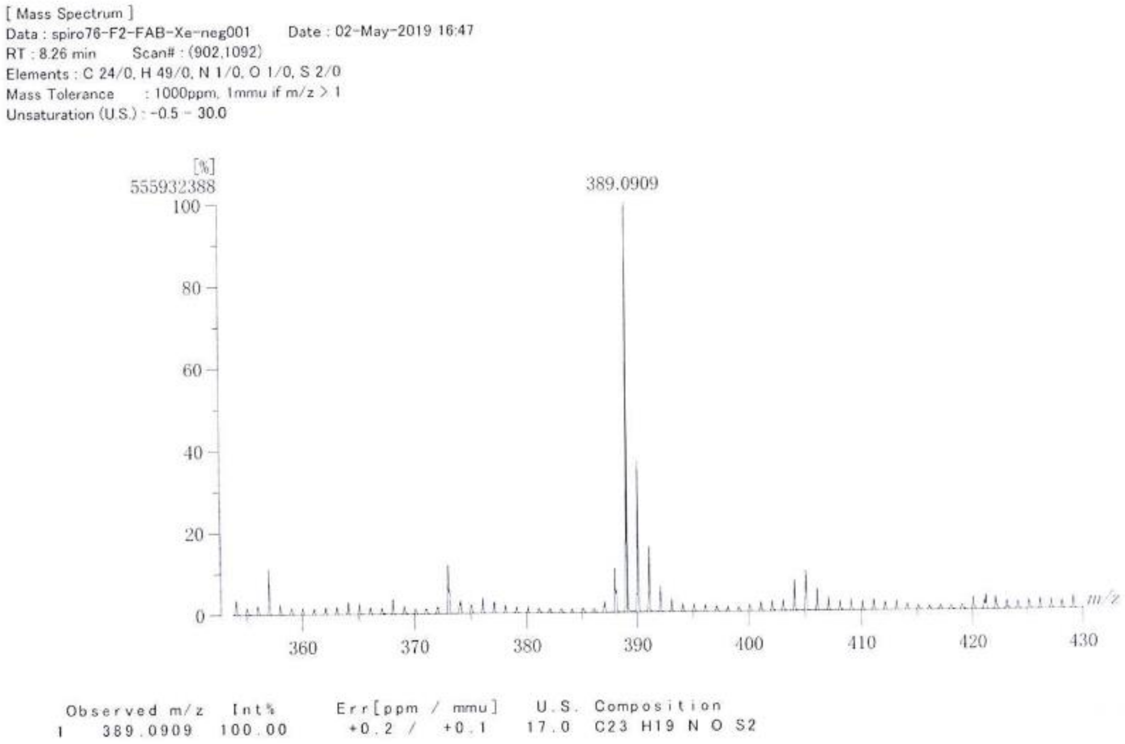

Supplementary Figure 8. HRMS of **T2**.

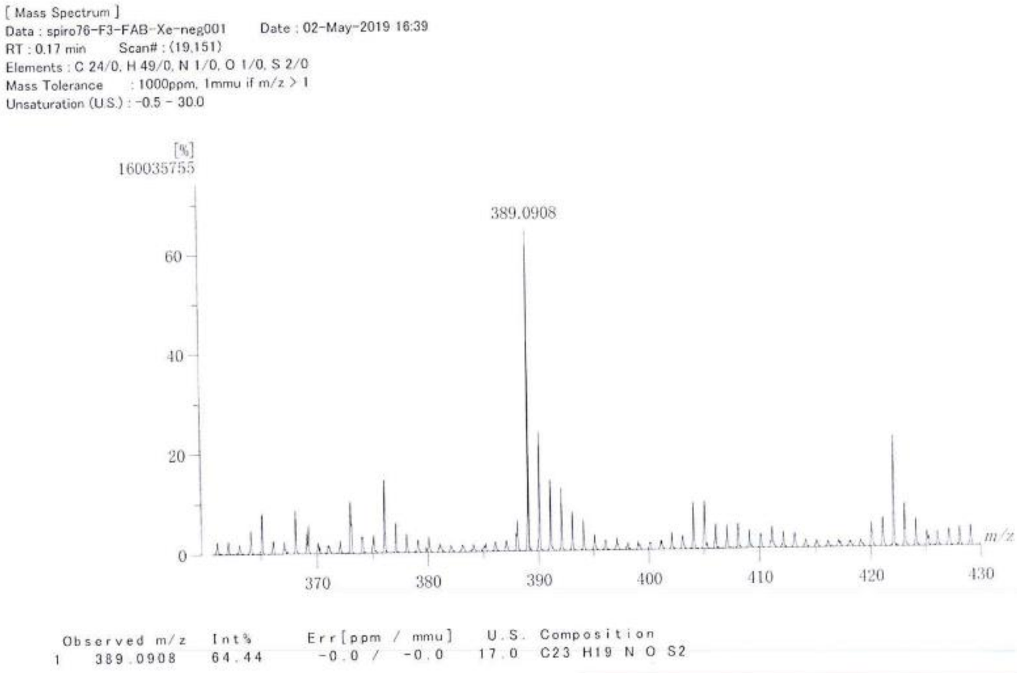

Supplementary Figure 9. HRMS of **T1**.

Comparative ^{1}H NMR spectra

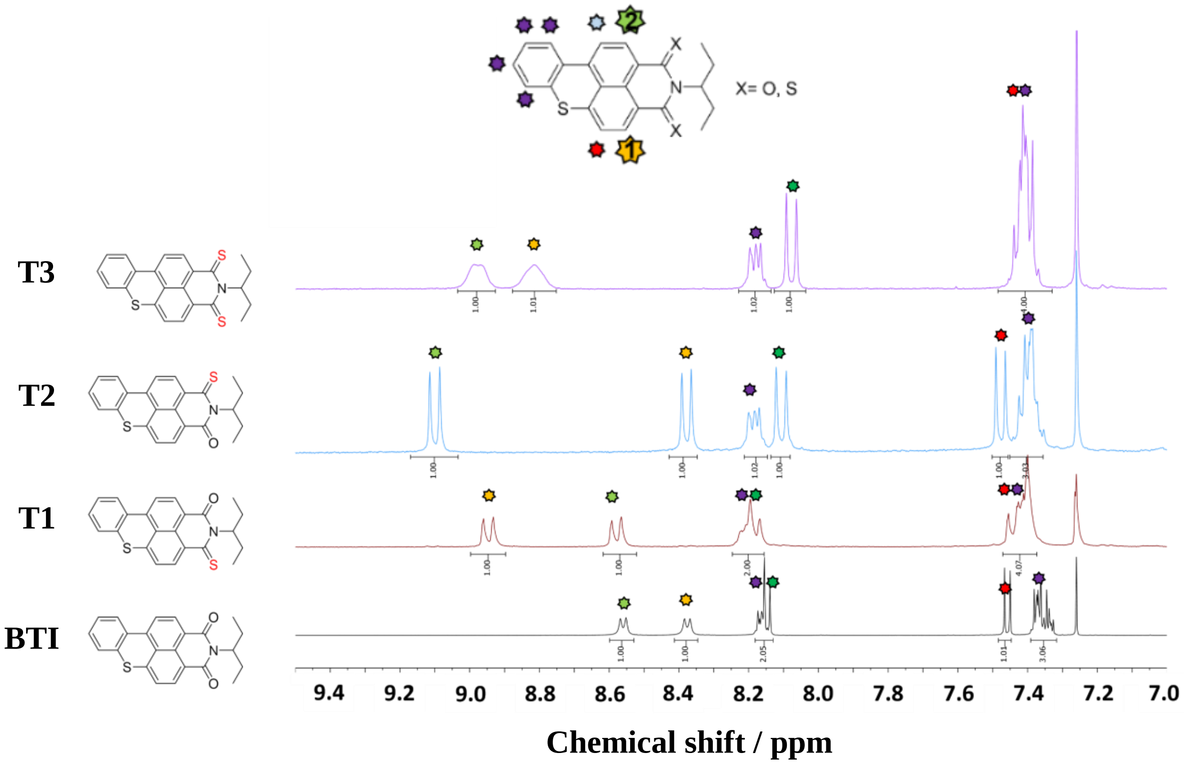

Supplementary Figure 10. Comparison of the ^{1}H NMR spectra recorded in $CDCl_3$ of the three isolated thionated derivatives and the reference **BTI**.

X-ray diffraction data

Supplementary Table 1. X-ray single-crystal diffraction data.

Crystal	**BTI**	**T1**	**T2**	**T3**
Formula	$C_{23}H_{19}NO_2S$	$C_{23}H_{19}NOS_2$	$C_{23}H_{19}NOS_2$	$C_{23}H_{19}NS_3$
Molecular Weight	373.45	389.51	389.51	405.57
Temperature (K)	295	293	293	150
Wavelength (Å)	1.54184	0.71073	0.71073	1.54184
Crystal system	Triclinic	Triclinic	Monoclinic	Triclinic
Space group	*P*-1	*P*-1	$P2_1/c$	*P*-1
a (Å)	7.4198(5)	7.535(3)	12.422(1)	7.3156(4)
b (Å)	8.8854(5)	8.983(3)	17.422(2)	8.1524(3)
c (Å)	15.1195(9)	15.745(4)	8.920(1)	31.566(1)
α (°)	101.609(5)	97.72(2)	90	83.411(3)
β (°)	90.283(5)	98.99(4)	103.64(1)	84.963(4)
γ (°)	111.324(5)	112.77(4)	90	84.884(3)
V ($Å^3$)	906.2(1)	948.1(6)	1876.0(3)	1857.0(1)
Z	2	2	4	4
Crystal color and form	orange prism	red plate	red plate	Brown needle
Crystal size (mm^3)	0.22x0.14x0.06	0.30x0.12x0.02	0.35x0.15x0.03	0.14x0.04x0.02
D_c (g cm^{-3})	1.369	1.364	1.379	1.451
F(000)	392	408	816	848
μ (mm^{-1})	1.728	1.364	0.297	3.697
Transmission (min/max)	0.2758/1.0000	0.872/0.993	0.803/0.992	0.9288/1.0000
θ (min/max) (°)	5.476/76.310	2.517/27.519	3.407/27.526	2.827/72.241
Data collected	6684	16616	26187	13425
Data unique	3620	4319	4309	6970
Data observed	3240	1523	2128	5428
R (int)	0.0329	0.1401	0.0893	0.0466
Nb of parameters	246	246	246	491
R_1 [I > 2σ(I)]	0.0644	0.0719	0.0515	0.0584
wR_2 [I > 2σ(I)]	0.1867	0.1356	0.1115	0.1360
R_1 [all data]	0.0681	0.2676	0.1495	0.0780

wR_2 [all data]	0.1955	0.1723	0.1418	0.1468
GOF	1.032	1.001	1.001	1.043
Largest peak in final: difference (e A^{-3})	0.551/-0.300	0.194 /-0.247	0.241/-0.234	1.093/-0.570
CCDC number	2083265	2116980	2116981	2116982

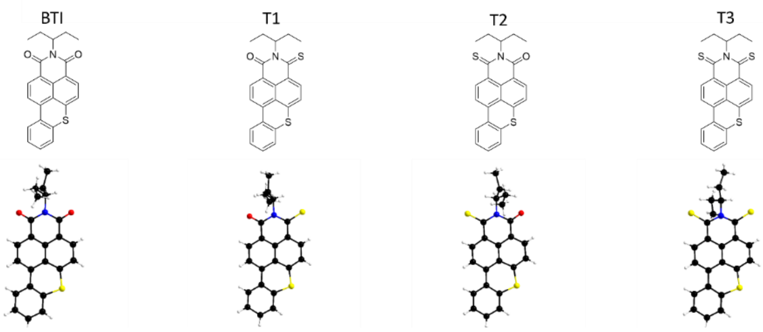

Distances / molecule	**BTI**	**T1**	**T2**	**T3**
C=O (Å)	1.223(2)<br>1.218(2)	1.235(4)	1.227(3)	-
C=S (Å)	-	1.664(4)	1.661(2)	1.656(3)<br>1.672(3)<br>1.651(3)<br>1.668(3)

Supplementary Figure 11. Structures and comparison of the C=O and C=S lengths from X-ray structures.

Singlet oxygen quantum yield

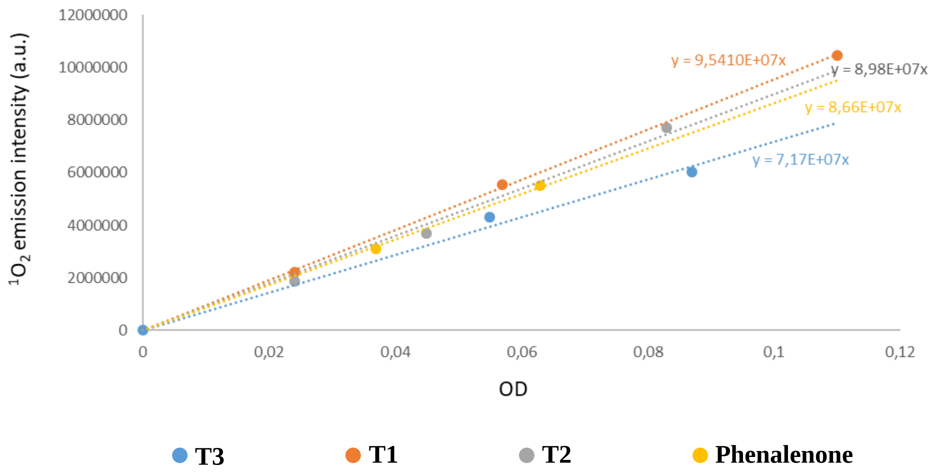

Supplementary Figure 12. Corrected singlet oxygen phosphorescence intensity vs optical density of the solution at the irradiation wavelength (λ_{ex}) of the reference and samples in $CDCl_3$. Linearization is featured as dotted line, with the corresponding slopes.

Photostability studies

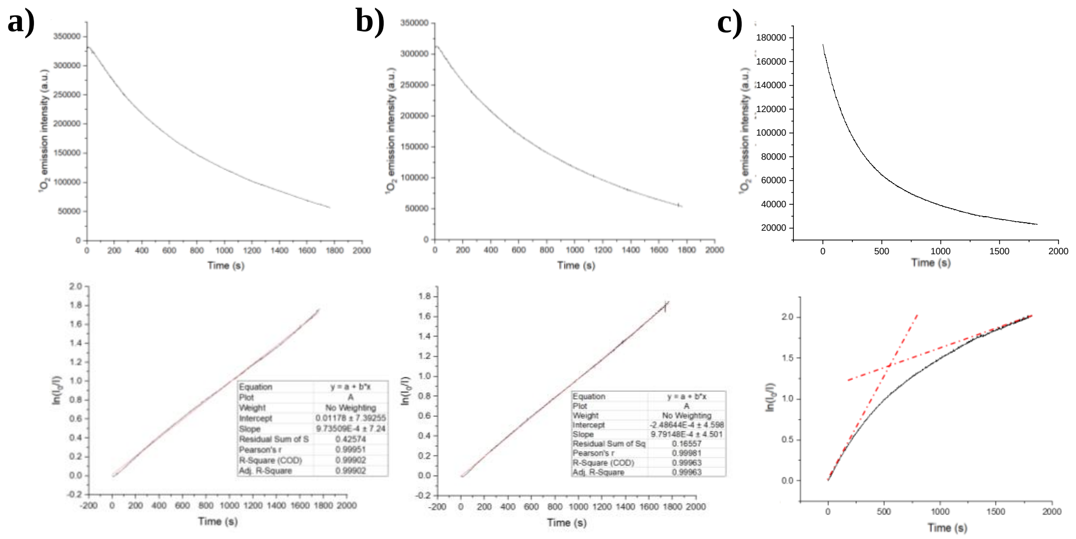

Supplementary Figure 13. Top panel: in-time following of the decrease of $^{1}O_2$ phosphorescence intensity (recorded at 1277 nm) upon continuous irradiation of 10^{-5} M solutions of **a) T1 b) T2** and **c) T3** in $CDCl_3$, at their respective maximal absorption wavelengths. Bottom panel: first-order plotting ($\ln(I_0/I)$ vs t ; I_0 and I stand for the emission intensity at t_0 and t, respectively, of this evolution for **a) T1 b) T2** and **c) T3** for **T1** and **T2** red lines correspond to linear fitting of the evolution, with fitting parameters featured as an inset; for **T3**, interrupted red lines are used here a visual guide to estimate the evolution rates at the beginning and end of the irradiation.

Photoconversion of T1, T2, T3 and BTI

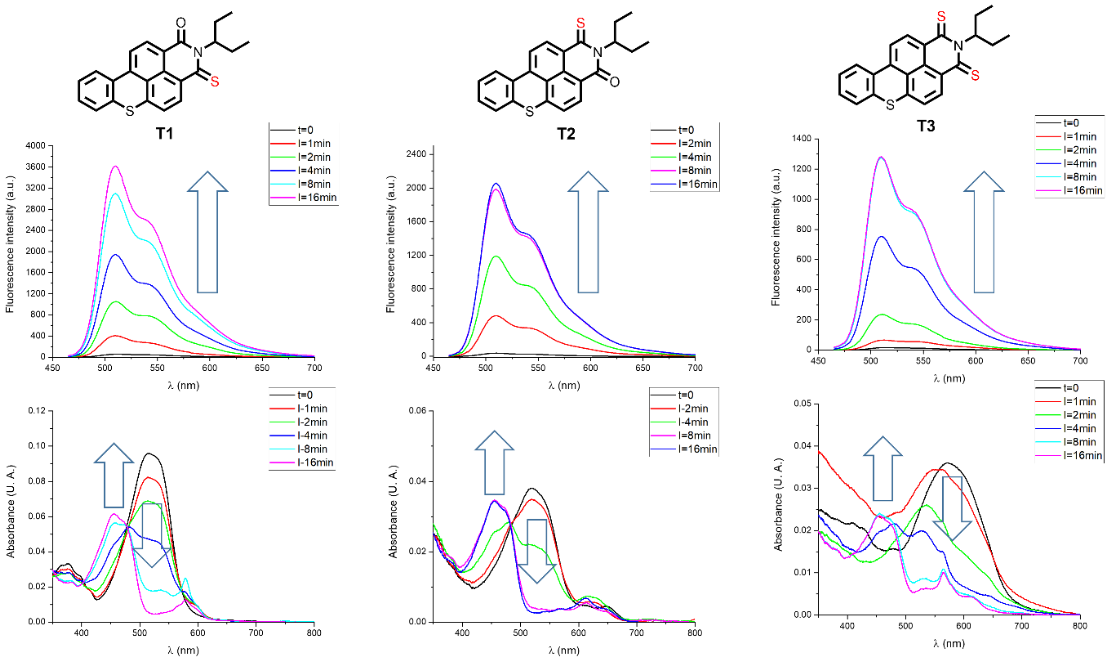

Supplementary Figure 14. Evolution of emission (top) and absorption spectra (bottom) of **T1**, **T2** and **T3** solubilized in non-deoxygenated dichloromethane under constant light irradiation.

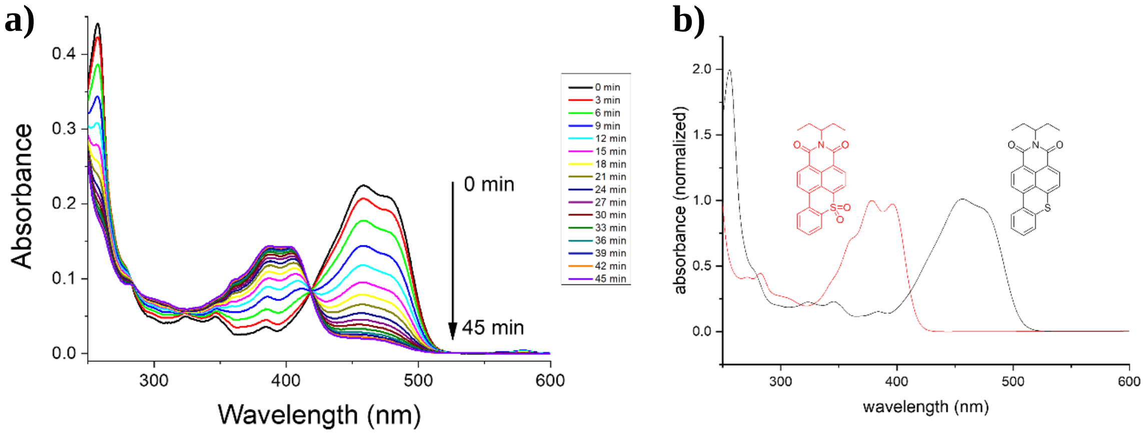

Supplementary Figure 15. a) Evolution of the absorption spectra of **BTI** solution in $CHCl_3$ (10 µM) under UV irradiation of 12W 254 nm lamp, t = 23 °C. **b)** Comparison between **BTI** and **BTI-SO$_2$** spectral signatures.

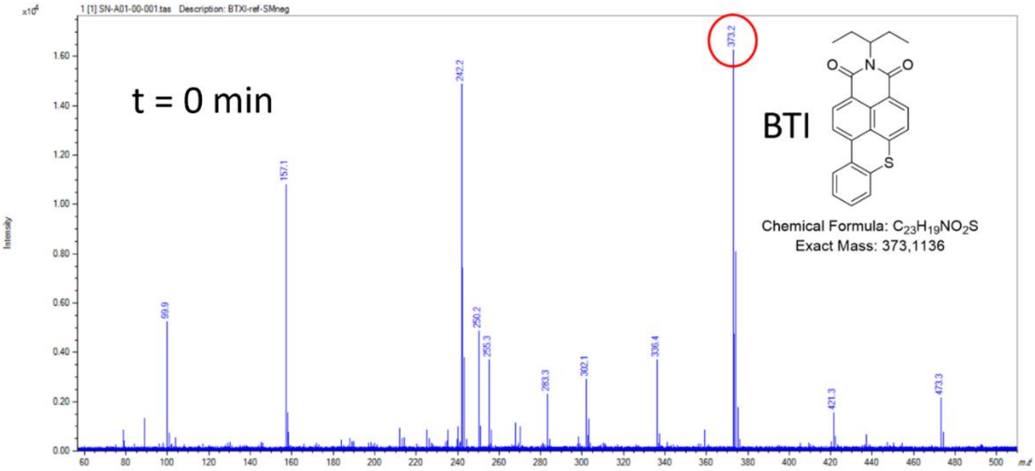

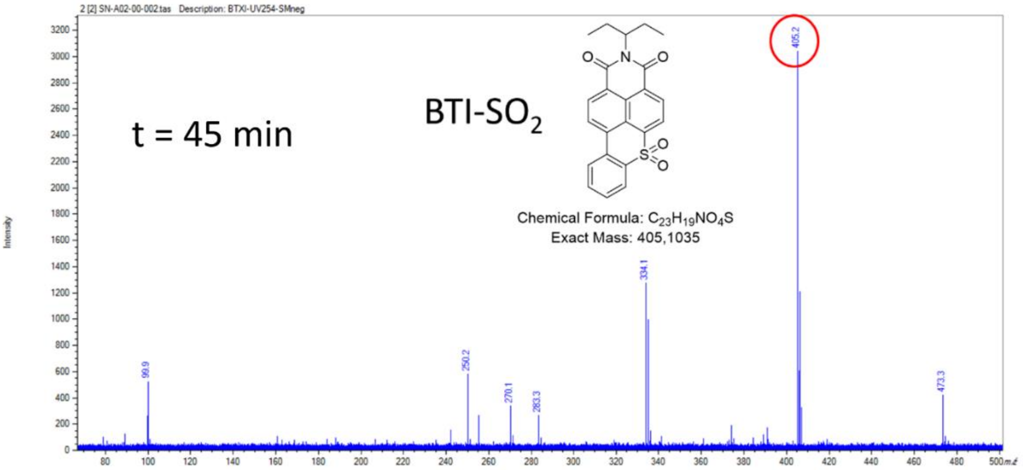

Supplementary Figure 16. Mass spectrum of aliquots collected from a non-deoxygenated $CHCl_3$ solution of **BTI** before and after UV light irradiation of 45 minutes.

EPR spectroscopy

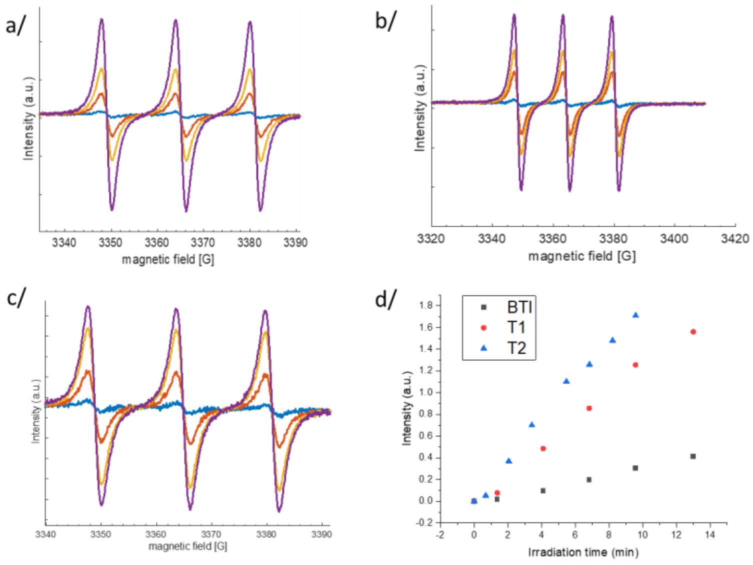

Supplementary Figure 17. Spin trap experiments in chloroform using TEMP as a singlet oxygen scavenger with **a) T1 b) T2** and **c) BTI** after 0 (blue line), 2 (red line), 5 (orange line) and 10 (purple line) minutes of irradiation at 530 nm. **d)** Comparison of the evolution of the intensity of TEMPO signal upon irradiation of **BTI**, **T1** and **T2**, in the abovementioned conditions.

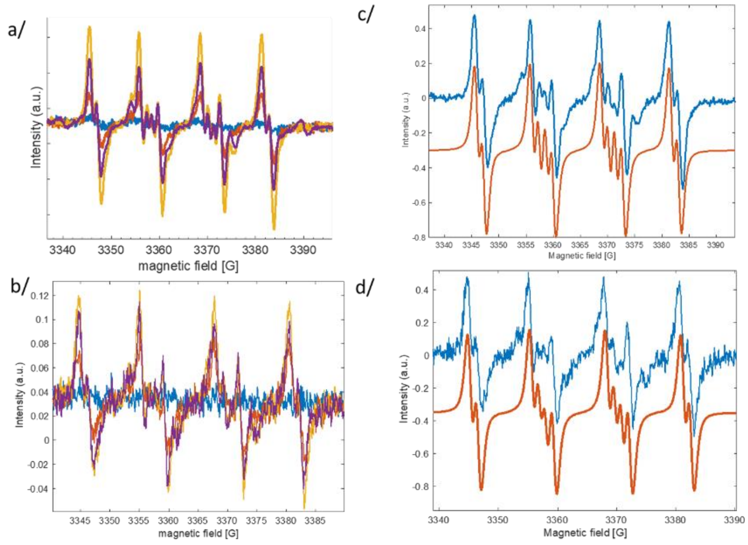

Supplementary Figure 18. Spin trap experiments in DMSO using DMPO as radical scavenger with **a) T1** and **b) BTI** after 0 (blue line), 1,5 (red line), 3 (orange line) and 4 (purple line) minutes of irradiation. Experimental (blue lines) and simulated (red lines) EPR spectra of **c) T1**, **d) BTI**, at 3 min irradiations in the same spin-trap conditions as for **a)** and **b)**.

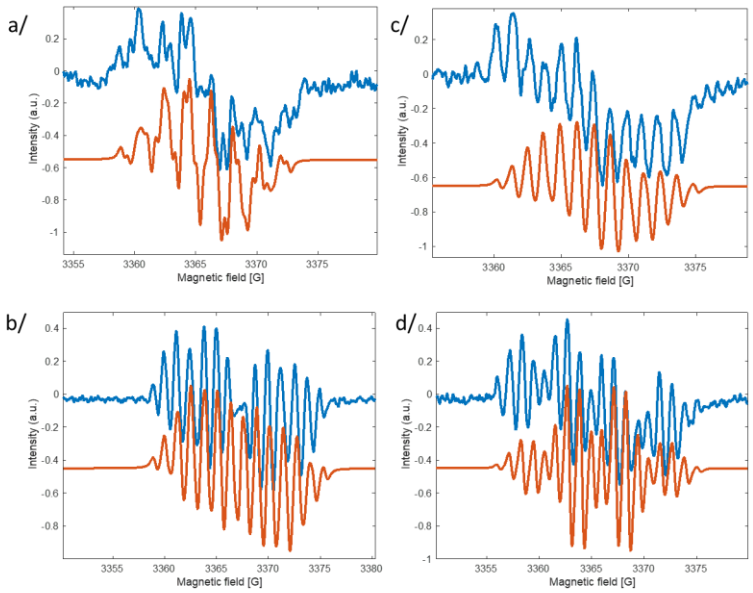

Supplementary Figure 19. Experimental (blue lines) and simulated (red lines) EPR spectra of **a) BTI**, **b) T1**, **c) T2**, **d) T3** in DMSO. Irradiation wavelengths are 455 nm (**BTI**) and 530 nm (**T1**, **T2** and **T3**).

Dynamic light scattering

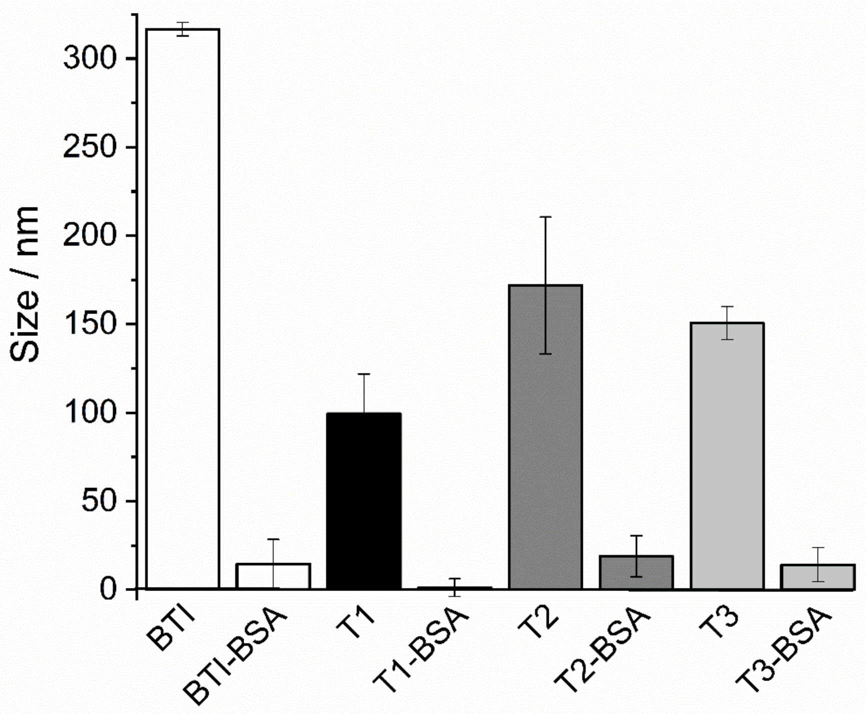

Supplementary Figure 20. DLS of compounds **BTI**, **T1**, **T2** and **T3** in the absence and presence of BSA in water. Compound concentration is in all cases 0.5 µM and BSA 50 µM. n = 3 independent experiments. Mean ± SD is indicated.

Photo-cytotoxic effect of T2 on HeLa cells upon green light irradiation

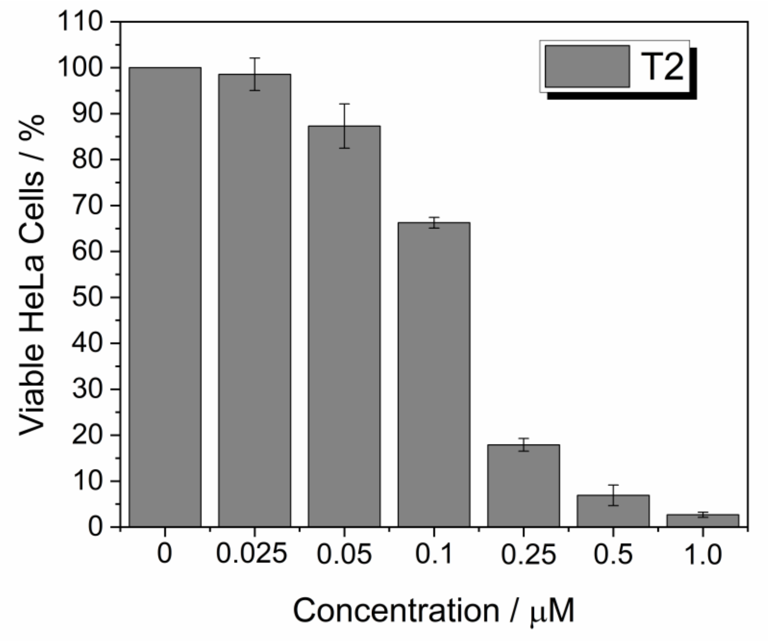

Supplementary Figure 21. Cytotoxic effects of **T2** on HeLa cells exposed to green light generated by a LED light cube (excitation = 542/20 nm, 10 mW cm^{-2}) for 6 min. n = 2 independent experiments. Mean ± absolute error is indicated.

Photo-cytotoxic effect of T3 on HeLa cells upon orange light irradiation

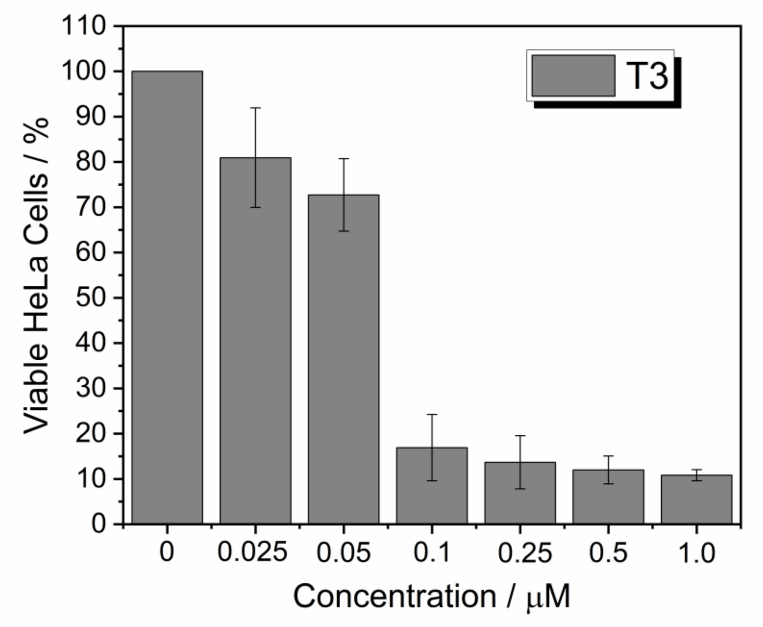

Supplementary Figure 22. Cytotoxic effects of **T3** on HeLa cells exposed to orange light generated by a LED light cube (excitation = 585/29 nm, 15.5 mW cm^{-2}) for 14 min. n = 2 independent experiments. Mean ± absolute error is indicated.

Green light-induced morphological changes of T2-treated HeLa cells

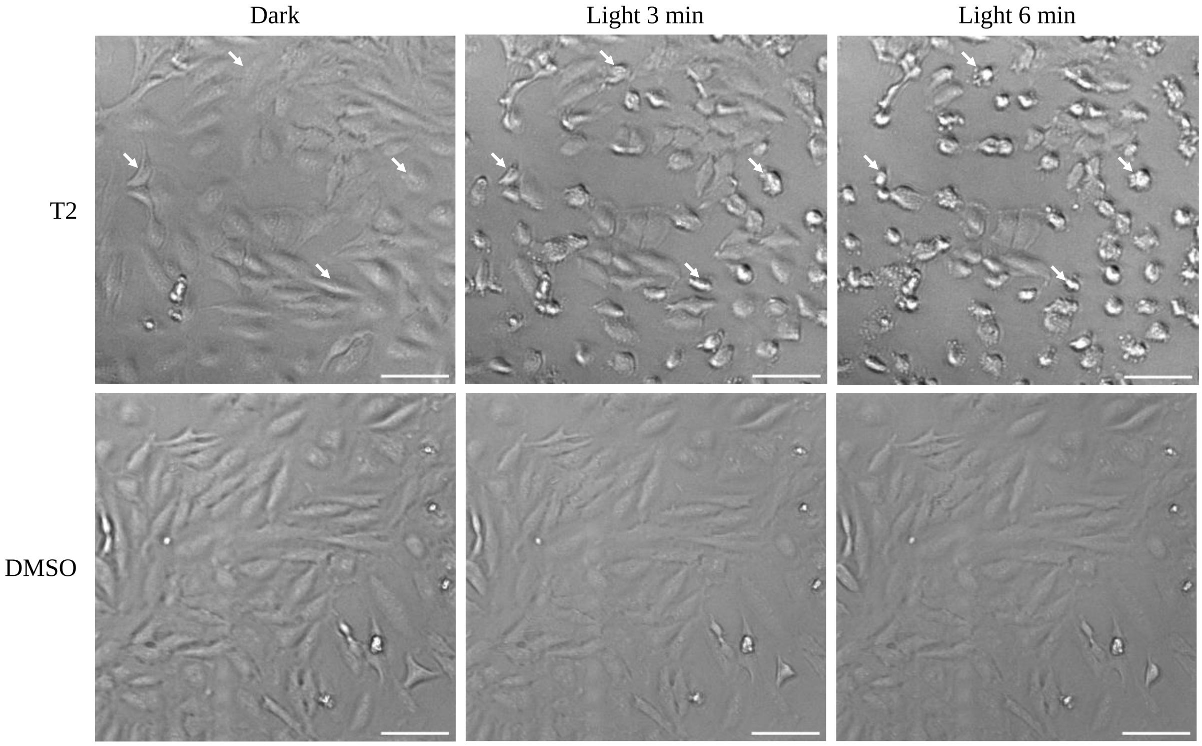

Supplementary Figure 23. Time-lapse experiments of live HeLa cells. Upper panel: light-induced morphological changes of **T2**-treated (1 μM) HeLa cells after 0, 3, and 6 min of green light irradiation. Lower panel: Control experiments performed in DMSO-treated HeLa cells. White arrows indicate different cells and how their morphology changes during the time-lapse. Scale bar = 100 μm.
